# *Ex vivo* infection model for *Francisella* using human lung tissue

**DOI:** 10.3389/fcimb.2023.1224356

**Published:** 2023-07-10

**Authors:** Kristin Köppen, Diana Fatykhova, Gudrun Holland, Jessica Rauch, Dennis Tappe, Mareike Graff, Kerstin Rydzewski, Andreas C. Hocke, Stefan Hippenstiel, Klaus Heuner

**Affiliations:** ^1^ Working group “Cellular Interactions of Bacterial Pathogens”, Centre for Biological Threats and Special Pathogens, Highly Pathogenic Microorganisms (ZBS 2), Robert Koch Institute, Berlin, Germany; ^2^ Department of Infectious Diseases, Respiratory Medicine and Critical Care, Charité - Universitätsmedizin Berlin, Corporate Member of Freie Universität Berlin and Humboldt-Universität zu Berlin, Berlin, Germany; ^3^ Advanced Light and Electron Microscopy, ZBS 4, Robert Koch Institute, Berlin, Germany; ^4^ Research Group Zoonoses, Bernhard Nocht Institute for Tropical Medicine, Hamburg, Germany; ^5^ Department for General and Thoracic Surgery, DRK Clinics, Berlin, Germany

**Keywords:** *Francisella*, intracellular bacteria, human lung, ex vivo, Tularemia, virulence

## Abstract

**Introduction:**

Tularemia is mainly caused by *Francisella tularensis* (*Ft*) subsp. *tularensis* (*Ftt*) and *Ft* subsp. *holarctica* (*Ftt*) in humans and in more than 200 animal species including rabbits and hares. Human clinical manifestations depend on the route of infection and range from flu-like symptoms to severe pneumonia with a mortality rate up to 60% without treatment. So far, only 2D cell culture and animal models are used to study *Francisella virulence*, but the gained results are transferable to human infections only to a certain extent.

**Method:**

In this study, we firstly established an *ex vivo* human lung tissue infection model using different *Francisella* strains: *Ftt* Life Vaccine Strain (LVS), *Ftt* LVS ΔiglC, *Ftt* human clinical isolate A-660 and a German environmental *Francisella* species strain W12-1067 (*F*-W12). Human lung tissue was used to determine the colony forming units and to detect infected cell types by using spectral immunofluorescence and electron microscopy. Chemokine and cytokine levels were measured in culture supernatants.

**Results:**

Only LVS and A-660 were able to grow within the human lung explants, whereas LVS ΔiglC and *F*-W12 did not replicate. Using human lung tissue, we observed a greater increase of bacterial load per explant for patient isolate A-660 compared to LVS, whereas a similar replication of both strains was observed in cell culture models with human macrophages. Alveolar macrophages were mainly infected in human lung tissue, but *Ftt* was also sporadically detected within white blood cells. Although *Ftt* replicated within lung tissue, an overall low induction of pro-inflammatory cytokines and chemokines was observed. A-660-infected lung explants secreted slightly less of IL-1β, MCP-1, IP-10 and IL-6 compared to *Ftt* LVS-infected explants, suggesting a more repressed immune response for patient isolate A-660. When LVS and A-660 were used for simultaneous co-infections, only the *ex vivo* model reflected the less virulent phenotype of LVS, as it was outcompeted by A-660.

**Conclusion:**

We successfully implemented an *ex vivo* infection model using human lung tissue for *Francisella*. The model delivers considerable advantages and is able to discriminate virulent *Francisella* from less- or non-virulent strains and can be used to investigate the role of specific virulence factors.

## 1 Introduction

*Francisella tularensis* is an intracellular Gram-negative bacterium causing tularemia, a life-threatening zoonotic disease occurring in various animals, including vertebrates, invertebrates and humans (Ellis et al., 2002; Foley and Nieto, 2010). More than 200 animal species have been identified to be susceptible for an infection by *Ft* and, therefore, *Ft* exhibits a broader host range than any other known zoonotic bacterial pathogen (Foley and Nieto, 2010; Santic et al., 2010). *Francisella* transmission to humans may occur via direct contact with infected animals (handling of infected animal, ingestion of insufficiently heated meat etc.), arthropod bites or through contaminated water or soil (Ellis et al., 2002; Maurin and Gyuranecz, 2016). The clinical manifestation of tularemia depends on the route of transmission and varies from flu-like symptoms to severe pneumonia with sometimes fatal outcome (Ellis et al., 2002; Maurin and Gyuranecz, 2016). The clinically most relevant *Francisella tularensis* subspecies are *Ft holarctica* (*Fth*) and *Ft tularensis* (*Ftt*). The latter is highly virulent (10-20 *Ftt* bacteria are sufficient to cause tularemia) and only occurs in North America (Saslaw et al., 1961a; Saslaw et al., 1961b; Ellis et al., 2002; Staples et al., 2006; Maurin and Gyuranecz, 2016). In contrast, being spread all over the Northern hemisphere *Fth* is moderately virulent, although 100-1000 *Fth* bacteria can already cause tularemia (Ellis et al., 2002; Bandouchova et al., 2009). However, infection with other *Francisella* species like *F. hispaniensis* (Whipp et al., 2003; Escudero et al., 2010), *F. novicida* (Hollis et al., 1989; Birdsell et al., 2009; Brett et al., 2014), *F. philomiragia* (Hollis et al., 1989; Wenger et al., 1989; Mailman and Schmidt, 2005; Kreitmann et al., 2015; Froböse et al., 2020) and *F. salimarina* (Hennebique et al., 2022) have been reported in immunocompromised patients. Due to its high infectivity and its ability to be spread by aerosols, *Ft* is classified as a potential bioterrorism agent of category A by the US Centers for Disease Control and Prevention (CDC) (Dennis et al., 2001; Maurin, 2015).

*Ft* has been shown to infect and replicate within various cell types including phagocytes (e.g. macrophages (Anthony et al., 1991), dendritic cells (Ben Nasr et al., 2006), neutrophils (Schwartz et al., 2012)) and non-phagocytic cells (e.g. fibroblasts (Horzempa et al., 2010) and epithelial cells (Melillo et al., 2006; Craven et al., 2008; Moreau and Mann, 2013)). For *Francisella* replication in mammals, macrophages serve as the major cell type (Twenhafel et al., 2009). After phagocytosis, *Francisella* prevents the fusion of the *Francisella*-containing phagosome with the lysosome and escapes into host cell cytosol, where a rapid bacterial replication culminates in cell lysis (Clemens et al., 2004; Bröms et al., 2010). During this process, the *Francisella* pathogenicity island, encoding a type VI secretion system (T6SS), is significantly involved (Lindgren et al., 2004; Santic et al., 2005; Bröms et al., 2010; Clemens et al., 2018). Deletion of the TSS6 tube structure protein IglC (corresponds to canonical TssD) results in a loss of phagosomal escape and intracellular replication culminating in an avirulent phenotype in mice (Golovliov et al., 1997; Golovliov et al., 2003; Lindgren et al., 2004; Santic et al., 2005).

To investigate *Francisella* infections *in vivo* different models have been established. For earlier studies, human volunteers were used (Mccrumb, 1961; Saslaw et al., 1961a; Saslaw et al., 1961b); later, different animal models were used, including mice (Bandouchova et al., 2009), rats (Kostiala et al., 1975; Ray et al., 2010) and non-human primates (Eigelsbach et al., 1962; White et al., 1964; Rick Lyons and Wu, 2007; Nelson et al., 2010; Glynn et al., 2015; Roberts et al., 2018). Also, often less pathogenic species, such as *F. novicida* or *Fth* live vaccine strain (LVS), were used to investigate *Francisella* virulence (Owen et al., 1964; Fortier et al., 1991; Rick Lyons and Wu, 2007; Hall et al., 2008). Although the course of infection by *Francisella* might be comparable between humans, animals and different *Francisella* strains, there are significant differences regarding host susceptibility and bacterial pathogenicity (Owen et al., 1964; Fortier et al., 1991; Rick Lyons and Wu, 2007; Hall et al., 2008). Hence, the transferability of the results obtained by infection using mice and a less virulent strain might be severely limited. In addition to the attenuated *Fth* LVS, we used an infectious wild-type *Fth* A-660 strain obtained from a patient suffering from lung tularemia (Appelt et al., 2019) in this study. As growing ethical concerns regarding the use of animal models emphasize the need for alternative experimental methods to examine *Francisella* infections, we established an *ex vivo* infection model using human lung tissue focusing on pulmonary tularemia representing a 3D model with different cell types. This *ex vivo* infection model has already been used for infection studies of different bacterial and viral pathogens like *Streptococcus pneumoniae* (Szymanski et al., 2012; Fatykhova et al., 2015; Berg et al., 2017; Peter et al., 2017), *Legionella pneumophila* (Jäger et al., 2014), *Haemophilus influenzae* (Wagner et al., 2015)*, Mycobacterium tuberculosis* (Ganbat et al., 2016), *Bacillus anthracis* (Booth et al., 2016), *Coxiella burnetii* (Graham et al., 2016), influenza A viruses H5N1 (Weinheimer et al., 2012), H5N8 (Grund et al., 2018), H7N9 (Knepper et al., 2013) and corona viruses (Hocke et al., 2013; Hönzke et al., 2022). The model allows quantification of pathogen replication and identification of cellular tropism, dissemination and tissue interaction including immune response. Here, we present an *ex vivo* infection model for *Francisella* using human lung tissue explants and diverse *Francisella* strains including an *Fth* wild-type isolate.

## 2 Materials and methods

### 2.1 Human lung tissues

Human lung explants were obtained from adult bronchial carcinoma patients (n = 10) undergoing lung resection at local thoracic surgery centers. Peripheral tumour-free lung tissue was used which is far from bronchial tumour and was resected during the surgical intervention for lung anatomical reasons. Written informed consent was obtained from all patients. The study was approved by the ethics committee of the Charité - Universitätsmedizin Berlin, Germany (project EA2/079/13) and performed in accordance with the approved guidelines. The healthy tissue was edited into small pieces (weight app. 0.1-0.2 g/piece) and cultivated in RPMI 1640 medium (RPMI) supplemented with or without 10% fetal calf serum (FCS; Merck, Darmstadt, Germany) at 37°C and 5% CO_2_, as described before (Fatykhova et al., 2015; Peter et al., 2017).

Motile and leached-out cells of human lung tissue explants were also used for infection studies. This cell suspension was obtained from the bottoms of sample containers and comprised mostly erythrocytes, lymphocytes and primary alveolar macrophages. Erythrocytes were lysed as described by Vuorte et al. (Vuorte et al., 2001). Briefly, the cell suspension was pelleted and resuspended in H_2_O for 15 sec. PBS was added, samples were centrifuged and the cell pellet was adjusted to 10^5^ cells/mL and seeded into 24-well plates. After over-night incubation cells were challenged with *Francisella* as described below.

### 2.2 Isolation of primary human alveolar macrophages and cell culture

Alveolar macrophages (AM) were isolated from human lung tissue as described above (Berg et al., 2017). Briefly, human lung tissue was repeatedly perfused with Hanks’ balanced salt solution (HBSS), and AM were seeded on glass coverslips in 12-well plates, 1x10^5^ cells/well in RPMI medium. After 4 h of adherence (37°C, 5% CO_2_) remaining erythrocytes were removed by repeated washing with HBSS. AM were cultured in RPMI medium supplemented with 2% FCS for 2 days.

The human macrophage-like cell line U937 (ATCC CRL-1593.2) and leached-out cells of human lung tissue (see above) were cultivated in RPMI medium supplemented with 10% FCS at 37°C and 5% CO_2_. Prior to the infection assays U937 cells were stimulated with PMA (phorbol-12-myristate-13-acetate, 1 mg/mL in dH_2_O [Sigma-Aldrich Chemie]) at a concentration of 1:20,000 for 36 h.

### 2.3 Bacterial strains and growth conditions

Strains used in this study are listed in **Table 1**. *Francisella* strains were cultivated in medium T (MT; (Pavlovich and Mishan'kin, 1987; Becker et al., 2016)) or on MT agar plates supplemented with hemoglobin and charcoal (MTKH plates, (Tlapak et al., 2018)).

**Table 1:** Strains used in this study.

Strain name	ID	Information	Reference
*Francisella tularensis* subsp. *holarctica* Live Vaccine Strain	*Fth* LVS	Derived from a virulent *Fth* isolate after diverse passages in mice; attenuated virulence in humans and mice (virulence is route and dose dependent in mice)	ATCC29684 (Tigertt, 1962)
*Francisella tularensis* subsp. *holarctica* LVS Δ*iglC*	*Fth* LVS Δ*iglC*	Mutant strain of *Fth* LVS, both *iglC* copies (essential part of FPI and *Francisella* T6SS) are deleted leading to absent intracellular replication and an avirulent phenotype in mice	(Golovliov et al., 2003)
*Francisella tularensis* subsp. *holarctica* human isolate A-660	*Fth* A-660	Obtained from patient suffering from pulmonic tularemia, isolated from blood culture	(Appelt et al., 2019)
*Francisella tularensis* subsp. *holarctica* human isolate A-271		Obtained from Eurasian beaver (*Castor fiber albicus*), isolated from lymph node	(Schulze et al., 2016)
*Francisella tularensis* subsp. *holarctica* human isolate A-663, A-820, A-981, A-1308		Human *Fth* isolates	(Appelt et al., 2019)
*Francisella* sp. isolate W12-1067	*F*-W12	German environmental strain obtained from water cooling system; FPI is absent, but several other virulence-associated genes are present	(Rydzewski et al., 2014)

### 2.4 Infection of human lung tissue and human cells

Human lung tissue explants were inoculated with 10^6^ CFU overnight grown *Francisella* strains for 2 h. Explants were washed three times with 2 mL plain RPMI and treated with 50 µg/mL gentamicin for 1 h to eliminate remaining extracellular bacteria. After washing them three-times and applying fresh plain RPMI, the lung explants were incubated up to 72 h. To determine the CFU/mL at various time points of the infection, human lung tissue was homogenized with Lysing Matrix D (MP Biomedicals) and saponin (0.001%; Sigma-Aldrich Chemie) in FastPrep-24 (MP Biomedicals) for 20 sec at 4 m/s and serial dilutions were plated on MTKH agar. Culture supernatants were collected and stored at -80°C until further analysis (see below).

For co-infection of *Fth* LVS and *Fth* A-660, human lung tissue explants were simultaneously challenged with both strains for 2 h (10^6^ (LVS) and 10^5^ (A-660) bacteria per explant). After washing and gentamicin treatment (same procedure as above), explants were homogenized and suspension was plated onto MTKH agar plates, half of which were supplemented with erythromycin (Ery), respectively, to distinguish between the two *Fth* strains. Belonging to biovar II *Fth* LVS is erythromycin-resistant, whereas *Fth* A-660, as a biovar I strain, is erythromycin-sensitive. Therefore, only *Fth* LVS grows on MTKH agar supplemented with Ery (MTKH+Ery; constituting *Fth* LVS CFU/mL). *Fth* A-660 CFU/mL values were calculated by subtracting CFU/mL gained from MTKH+Ery plates from total CFU/mL rates received on MTKH plates (on which both strains are able to grow).

For infection of human macrophages (AM, U937), cells were seeded at a concentration of 5 × 10^5^ cells/mL and challenged with overnight grown *Francisella* strains for 2 h (multiplicity of infection (MOI) of 10). After removing the bacterial suspension, cells were washed and treated with 50 µg/mL gentamicin for 1 h. Subsequently, plain medium was added and cells were incubated up to 72 h. To determine the CFU at various time points of infection, cells were lysed with saponin (0.001%), and serial dilutions were plated on MTKH agar.

For the co-infection assay, U937 cells were seeded at a concentration of 5 × 10^5^ cells/mL and infected with a mixture of *Fth* LVS and *Fth* A-660 bacteria (1:1; in a total MOI of 10) for 2 h. Cells were treated with 50 µg/mL gentamicin for 1 h, afterwards washed and incubated up to 72 h (same procedure as for infection of human macrophages, see above). At various time points, U937 cells were lysed with 0.001% saponin, and bacterial suspension was plated on MTKH agar plates, half of which were supplemented with erythromycin (Ery), respectively, to distinguish between the two *Fth* strains as described above.

### 2.5 Immunohistochemistry and confocal immunofluorescence

Isolated AM were challenged with *Fth* A-660 as described in section “Infection of human lung tissue and cells”. After 48 h AM were fixed as described before (Berg et al., 2017) and stained with an established cell marker for AM CD68 (abcam, Cambridge, UK) and anti-*Ft* LPS-FITC antibody (F11FITC, (Grunow et al., 2000)). Immunofluorescence of AM was analyzed using a LSM 780 [(objectives: Plan Apochromat 63x/1.40 oil DIC M27 and 40x/1.30 oil DIC M27), Carl-Zeiss, Jena, Germany].

For immunohistochemistry of infected human lung tissue, explants were challenged with 10^6^ bacteria/mL for 24 h and 48 h. Afterwards the samples were fixed in 3% paraformaldehyde for 48 h, embedded in paraffin and routinely processed for histology and immunofluorescence staining as described before (Peter et al., 2017; Hönzke et al., 2022). For characterization of infected cells, the specific cell marker CD68 for AM and anti-*Ft* LPS antibody (Grunow et al., 2000) were used, followed by incubation with corresponding secondary antibodies. Nuclei were subsequently counterstained with DAPI (Sigma Aldrich). Immunofluorescence of human lung slices was analyzed by spectral confocal microscopy using a LSM 780 [(objectives: Plan Apochromat 63x/1.40 oil DIC M27 and Plan Apochromat 40x/1.40 oil DIC M27), Carl-Zeiss, Jena, Germany]. Based on a spectral image lambda stack, linear unmixing of tissue autofluorescence and overlapping spectra of fluorochromes were performed using ZEN 2012 software (Carl-Zeiss, Jena, Germany). To reveal lung and cell morphology, images were combined with Differential Interference Contrast (DIC). All image sets were acquired using optimal configuration regarding resolution and signal to noise ratio. Images were processed using ZEN 2012.

### 2.6 Electron microscopy

Human lung explants were challenged with 10^8^ *Francisella* bacteria per mL for up to 48 h. Electron microscopy of human lung explants was done essentially as described before (Weinheimer et al., 2012). Lung tissue was fixed by immersion in 4% Formaldehyde, 2.5% glutaraldehyde (in 50 mMHepes-buffer) and post fixed with 1% OsO4 (1 h), 0.1% tannic acid (in 50 mM Hepes buffer, 30 min) and 2% uranyl acetate (2 h). Samples were dehydrated in ethanol and embedded in epon resin. Thin sections were produced using an ultramicrotome (UC7, Leica, Wetzlar, Germany) and stained with 2% uranyl acetate (20 min) followed by lead citrate (2 min). Sections were examined using a transmission electron microscope (Tecnai Spirit, Thermo Fisher/FEI) operated at 120 kV. Images were recorded using a CCD-camera (Phurona, Emsis, Münster, Germany).

### 2.7 Cytokine and chemokine measurement

Supernatants of infected human lung tissue explants were collected 24 h, 48 h and 72 h post infection. After centrifugation (10 min, 5000 g, 4°C) 100 µl aliquots were stored at liquid nitrogen until further investigations. Samples were sterilized using 0.2 µm filter (Sartorius, Göttingen, Germany) prior to measurement of cytokines and chemokines. Cytokines and chemokines were analyzed in cell culture supernatants using the LegendPlex assay (BioLegend, USA) according to the manufacturer’s instructions. For the analyzed biomarkers, the detection limits of the LegendPlex assay were, as follows: granulocyte colony-stimulating factor (G-CSF, 33.66 pg/mL), interferon-α (IFNα; 3.11 pg/mL), IFNγ (3.35 pg/mL), interleukin (IL) 1ß (4.23 pg/mL), IL-2 (1.93 pg/mL), IL-4 (2.22 pg/mL), IL-5 (2.7 pg/mL), IL-6 (3.09 pg/mL), IL-8 (2.53 pg/mL), IL-9 (3.12 pg/mL), IL-10 (1.9 pg/mL), IL-12p70 (4.75 pg/mL), IL-13 (3.93 pg/mL), IL-17A (3.39 pg/mL), IL-17F (2.07 pg/mL), IL-21 (4.04 pg/mL), IL-22 (2.16 pg/mL), interferon-γ–induced protein-10 (IP-10 = C-X-C motif chemokine ligand 10 (CXCL-10), 4.48 pg/mL), monocyte chemotactic protein-1 (MCP-1 = CC-chemokine ligand 2 (CCL-2); 6.04 pg/mL), tumor necrosis factor-α (TNFα, 2.41 pg/mL) and vascular endothelial growth factor (VEGF, 31.41 pg/mL).

### 2.8 Statistics

Statistical analysis was performed with GraphPad Prism 9 software. For comparison between the two analyzed groups (LVS vs. A-660), a two-tailed test *t* was used. For comparison of three groups (LVS vs. A-660 and A-660 10^5^, respectively), one-way ANOVA and Kruskral-Wallis test with multiple comparison were performed.

## 3 Results

### 3.1 Establishment of the human *ex vivo* infection model for *Francisella*

In order to determine the growth of intracellular *Francisella*, represented by the colony forming units (CFU), the human lung explants needed to be homogenized and lysed after infection. To test if *Francisella* is able to survive the homogenization, 10^5^ *Fth* LVS bacteria were treated in PBS supplemented with 0.001% saponin for 20 sec with 4 m/s using a FastPrep homogenizer. As shown in **Figure S1A**, the homogenization with FastPrep did not significantly reduce the bacterial load of the LVS compared to untreated and vortexed samples. In order to monitor a possible extracellular replication of *Francisella*, we used *Fth* LVS Δ*iglC* in all infection assays as a control strain. This mutant strain is unable to escape the phagosome and is therefore, unable to replicate intracellularly (Golovliov et al., 1997; Golovliov et al., 2003; Lindgren et al., 2004; Santic et al., 2005). When U937 macrophages were infected with LVS and LVS Δ*iglC* for 2 h, washed and incubated for 24 h and 48 h, respectively, CFU/mL of both, LVS and LVS Δ*iglC*, increased over time (**Figure S1B**), demonstrating that *Fth* replicates in co-culture with U937 cells. Treatment by gentamicin inhibited the extracellular replication (**Figure S1B**). After observing a minimal increase of bacteria (OD_600nm_) cultivated in RPMI + 10% FCS (data not shown), for all following infection assays, RPMI medium was used without FCS to minimize a possible extracellular replication of *Fth* in our model.

**Figure 1:**
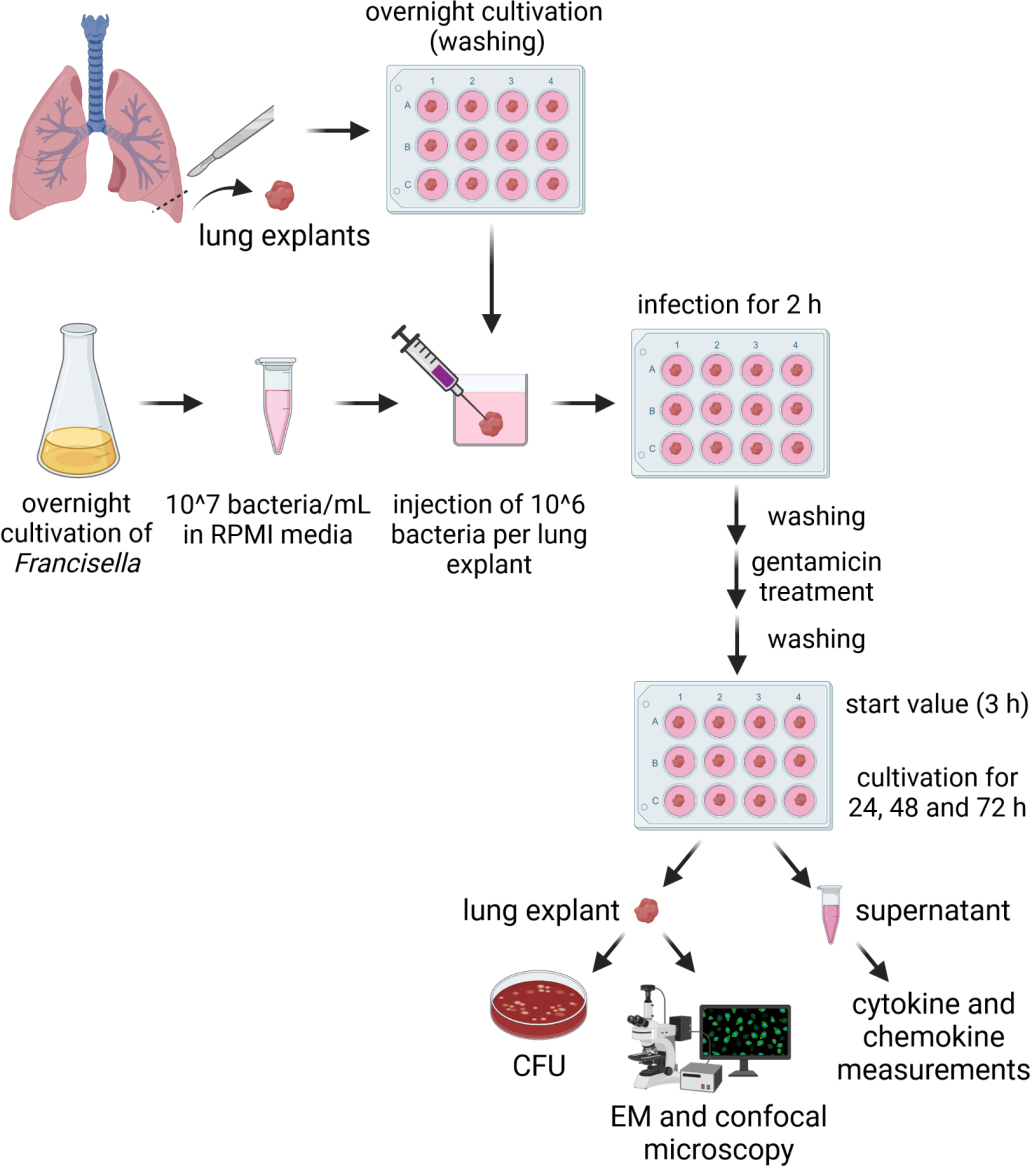
Scheme of the human lung *ex vivo* infection model for *Francisella*. 10^6^ *Francisella* bacteria were injected into human lung tissue explants portioned in three punctures at different lung tissue sections (~ 33 µl each) and incubated for 2 h at 37°C and 5% CO_2_. Bacterial suspension was completely removed; lung explants were washed and treated with 50 µg/mL gentamicin for 1 h to eliminate remaining extracellular bacteria. Medium was removed; lung explants were washed and fresh plain RPMI was added. Human lung tissue was used to determine the number of intracellular bacteria and to identify the infected cell types using immunofluorescence and electron microscopy. Culture supernatants were used to measure cytokine and chemokine levels. Schema was created with BioRender.com (https://biorender.com).

The experimental procedure of the *ex vivo* infection model was adapted for *Francisella*, as follows (**Figure 1**): 10^6^ *Francisella* bacteria were injected into human lung tissue explant portioned into three punctures (~ 33 µl each). The infection was performed at 37°C and 5% CO_2_ for 2 h. Medium was completely removed; lung explants were thoroughly washed and treated with 50 µg/mL gentamicin for 1 h to eliminate the remaining extracellular bacteria. Subsequently, medium was removed, followed by a complete rinsing of explants. Afterwards, fresh plain RMPI medium was added and the lung explants were incubated for up to 72 h. The human lung tissue was further used (1) to determine the intracellular growth and (2) to identify the infected cell types. To achieve this, (1) human lung explants were homogenized, lysed and plated on agar plates to determine the CFU per g lung tissue (**Figure 1**). (2) The analysis by differential interference contrast and electronic microscopy was conducted after fixing the human lung explants and staining them with antibodies for *Fth* as well as selected cell markers.

### 3.2 Multiplication of *Francisella* strains in human lung explants.

**Figure 2:**
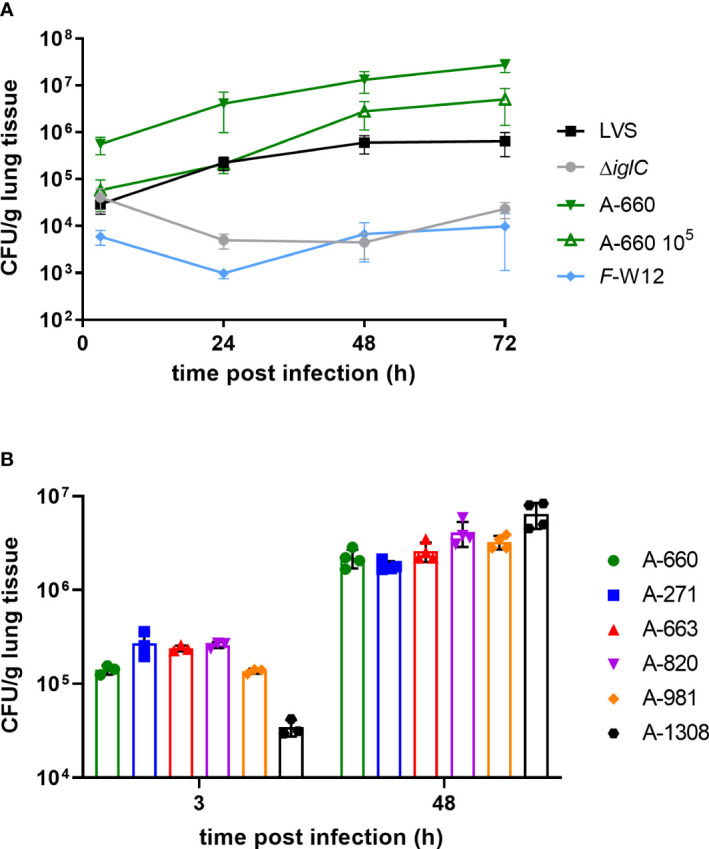
*Francisella* human lung *ex vivo* infection model. **(A)** Lung tissue explants were infected with 10^6^ *Fth* LVS, *Fth* LVS Δ*iglC*, *Fth* wild-type A-660 and *F*-W12 bacteria using experimental procedure described in legend of **Figure 1** and in material and methods. After 3 h, 24 h, 48 h and 72 h of infection human lung tissue was homogenized. Bacterial suspension was plated onto agar plates to obtain the colony forming units (CFU) per g lung tissue. Human lung explants were additionally infected with 10^5^ bacteria of *Fth* wild-type A-660 (A-660 10^5^). Means with structural equation modeling of at least five individual experiments are shown. A one-way ANOVA and Kruskral-Wallis test with multiple comparisons were used to compare LVS vs. A-600 and A-660 10^5^, respectively. For comparison of LVS vs. A-600 significances were observed (3 h: p = 0.0076; 24 h: p = 0.0110; 48 h: p = 0.0076; 72 h: p = 0.0047), but comparison of LVS with A-660 10^5^ remained statistically insignificant. **(B)** Lung tissue explants were infected with six German *Fth* isolates (A-#). Means with standard deviation of one experiment are shown.

In this study we used *Fth* LVS, *Fth* LVS Δ*iglC* and a human isolate *Fth* (*Fth* A-660) obtained from a tularemia patient exhibiting pneumonia (Appelt et al., 2019), as well as an environmental *Francisella* species (*Francisella* sp. strain W12-1067, *F*-W12) isolated from a cooling tower in Germany (Rydzewski et al., 2014). It is yet not known if the environmental species *F*-W12 is pathogenic for humans, but the species possesses some well-known virulence factors of *Francisella* and is able to persist in mouse macrophages and *Acanthamoeba lenticulata* (Rydzewski et al., 2014; Köppen et al., 2019). By using the human lung *ex vivo* infection model, an increase of CFU/g could be shown in the lung explants over 72 h for *Fth* LVS and *Fth* wild-type A-660. During this process, it became obvious that the human isolate *Fth* A-660 replicated to a greater extent than *Fth* LVS leading to a higher CFU count per g lung tissue (**Figure 2A**). Over a time period of 72 h, a 22-fold CFU-increase/g tissue was observed for *Fth* LVS and a 49-fold increase for A-660 (A-600: 2.7 × 10^7^ CFU/g; LVS: 2.4 × 10^5^ CFU/g; p = 0.0047). A similar trend was obtained when a lower bacterial load of *Fth* A-660 was used (10^5^ bacteria instead of 10^6^ per lung explant). Here, we observed an 86-fold CFU-induction/g lung tissue (A-660 10^5^ after 72 h: 5 × 10^6^ CFU/g, LVS: 2.4 × 10^5^ CFU/g; p = 0.3537). In contrast, the bacterial load of *Fth* LVS Δ*iglC* did not increase in lung explants over time, instead a minor reduction was obtained (0.56-fold induction from 24 h to 72 h, **Figure 2A**). Neither replicated the environmental *Francisella* strain *F*-W12 in the human lung explants, but the strain persisted more or less stable over a time period of 72 h (1.6-fold induction; **Figure 2A**). Hence, the human lung *ex vivo* infection model confirmed the theoretically expected intracellular growth of different *Francisella* strains. To further underpin the reliability of the observed results, five other *Fth* isolates (one animal isolate obtained from a Eurasian beaver: A-271; four human isolates obtained from tularemia patients: A-663, A-820, A-981, A-1308) were investigated. All tested isolates showed an increase of CFU per g lung tissue comparable to those observed for *Fth* A-660 after 48 h (**Figure 2B**).

**Figure 3:**
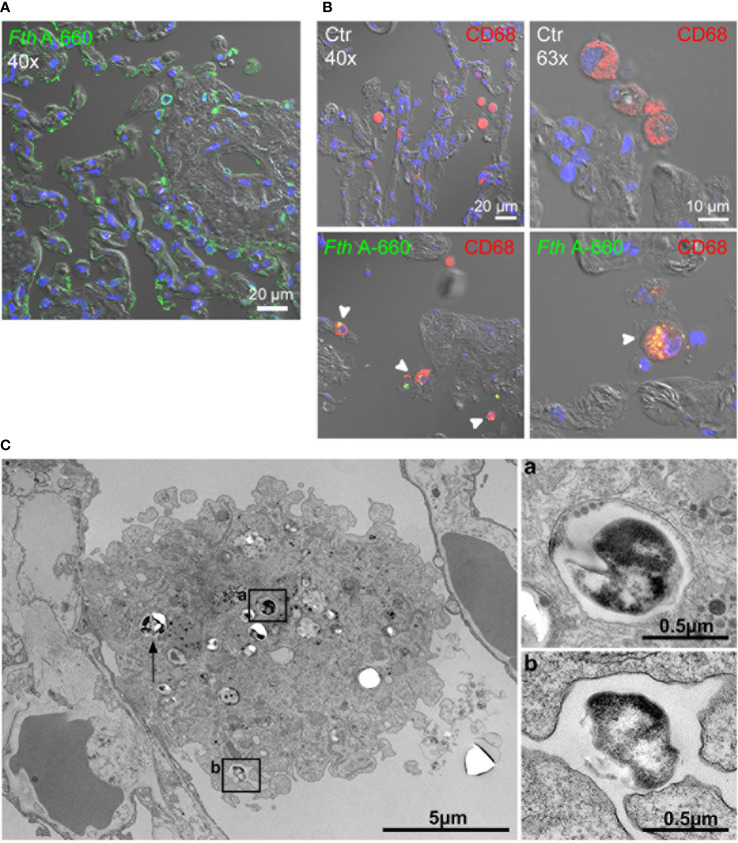
*Fth* wild-type A-660 (*Fth* A-660) distribution in human lung tissue explants 48 h post infection. **(A, B)** Confocal immunofluorescence microscopy of sections from infected lung explants. *Fth* A-660 bacteria are shown by binding of an antibody against LPS (green), nuclei were counterstained with DAPI (blue) and the tissue was visualized by differential interference contrast. **(A)** At the putative injection spot of bacteria into the lung explant, the green signal for bacteria is associated with the epithelial lining of the alveoli, the endothelium of a small vessel and within a few cells in the extracellular space and the connective tissue. **(B)** A region distinct from the putative injection spot, signals for bacteria (green) were mainly found in alveolar macrophages (red; CD68) **(C)** Electron microscopy of thin sections from infected lung explants shows a macrophage in the alveolar space with contact to the epithelial lining of an alveolus. Two bacterial profiles (box a and arrow) are localized in the cytoplasm of the macrophage within a membrane-bound compartment. One bacterium is associated with a surface niche of the macrophage (box b).

We further aimed to identify the cell types involved in the course of a *Francisella* infection of the human lung by applying spectral immunofluorescence to *Fth* A-660-infected lung explants. After 48 h, parts of highly infected areas were found. Especially, the assumed injection spot showed a high concentration of *Fth* A-660 bacteria, as shown in **Figure 3A**. Apart from the injection sites, *Fth* A-660 was primarily detected in human alveolar macrophages in distinct areas (see **Figure 3B**). These results were confirmed by examining *Fth* A-660 infected lung explants by electron microscopy (**Figure 3C**). However, *Fth* A-660 was not only detected in alveolar macrophages, but also sporadically in other cell types, including lymphocytes, granulocytes and fibrocytes, as well as, extracellular and within the connective lung tissue (**Supplemental Figure S2**).

**Figure 4:**
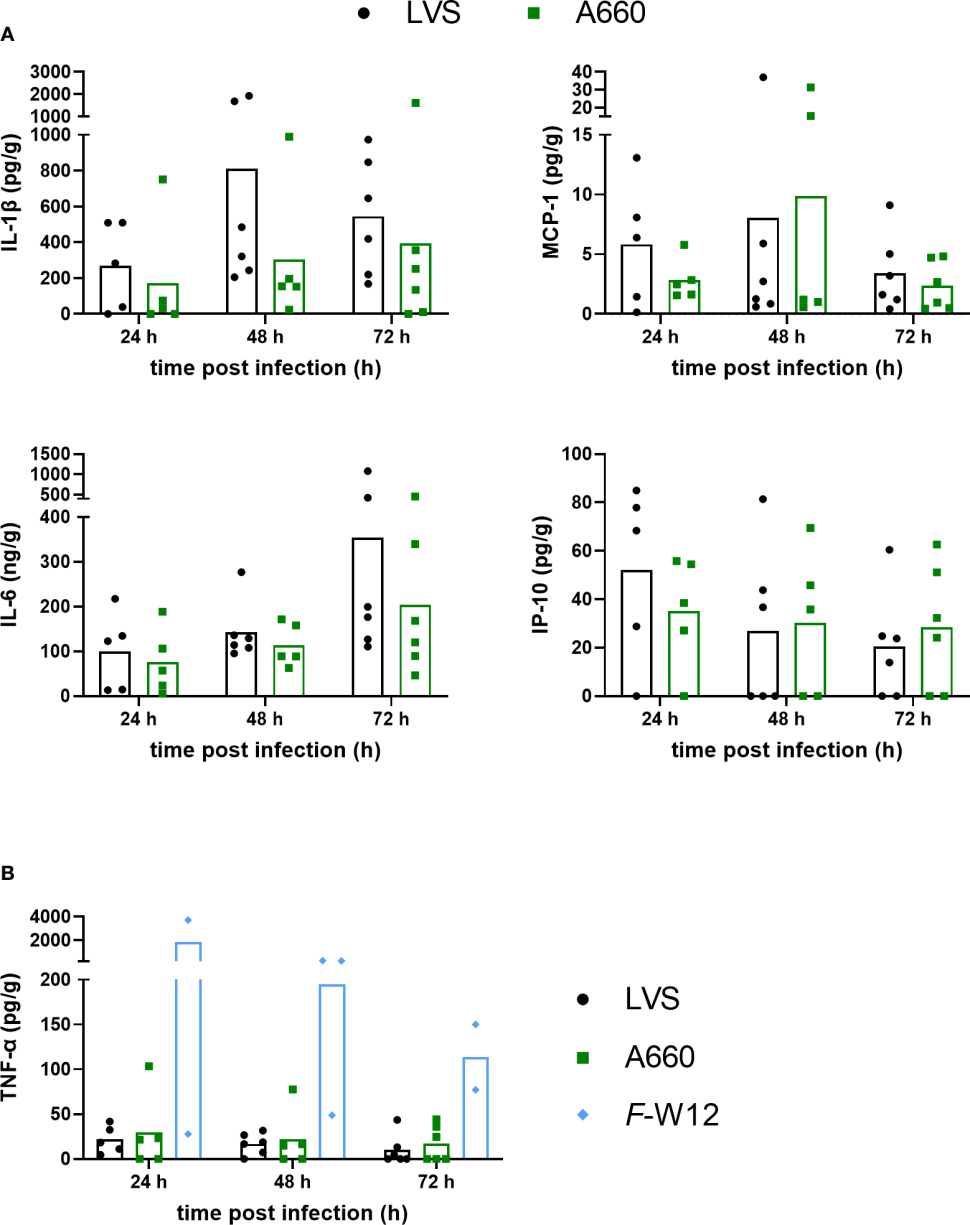
Cytokine levels obtained using the *Francisella* human lung *ex vivo* infection model. Lung tissue was infected with A-660 and LVS **(A)** A-660, LVS and *F*-W12 **(B)** using experimental procedure as described in the legends of **Figures 1** and **2**. After 24 h, 48 h and 72 h of infection, the supernatants were collected and the levels of IL-1β, MCP-1, IL-6, IP-10 **(A)** and TNF-α **(B)** were measured. Means are indicated.

As a next step, we assessed how *Francisella* activated the host response in *ex vivo* infected human lungs by quantifying the induced cytokine and chemokine profile. This was performed by collecting and analyzing the culture supernatants of lung explants infected by *Fth* LVS, *Fth* LVS Δ*iglC*, *Fth* A-660 and *F*-W12 24 h, 48 h and 72 h post infection to determine the levels of cytokines and chemokines (e.g. IL-1β, IL-6, TNF-α, IL-8, G-CSF, MCP-1, IP-10 and VEGF). Just as the individual immune response differs from human to human, the induced cytokine and chemokine production varied between *ex vivo* infected lung explants (see **Figure S2**) and remained not to be statistically significant. Nevertheless, the following tendencies could be observed: The cytokine and chemokine response differed between *Fth* A-660 and *Fth* LVS. *Fth* A-660 seemed to induce a lower secretion of IL-1β (p = 0.0519 after 48 h), MCP-1 and IL-6 compared to *Fth* LVS (**Figure 4**). After 24h, a lower concentration of IP-10 seemed to be induced by A-660, but after 72 h, a higher concentration of IP-10 was detected in A-660-infected human lung explant supernatants compared to LVS (**Figure 4**). We did not identify distinctive differences in secretion of IL-8, G-CSF and VEGF between *Fth* LVS and *Fth* A-660 infected lung explants (**Figure S2**). In general, the environmental *F*-W12 strain seemed to induce higher levels of cytokines, including TNF-α, in the infected lung explants (**Figures 4**, **S2**).Various further chemokines and cytokines, including IL-2, IL-4, IL-5, IL-9, IL-10, IL-12p70, IL-13, IL-17A, IL-17F, IL-21, IL-22, IFN-α and IFN-γ, were investigated but remained below the detection limits (data not shown).

### 3.3 Replication of *Francisella* strains in cell culture

**Figure 5:**
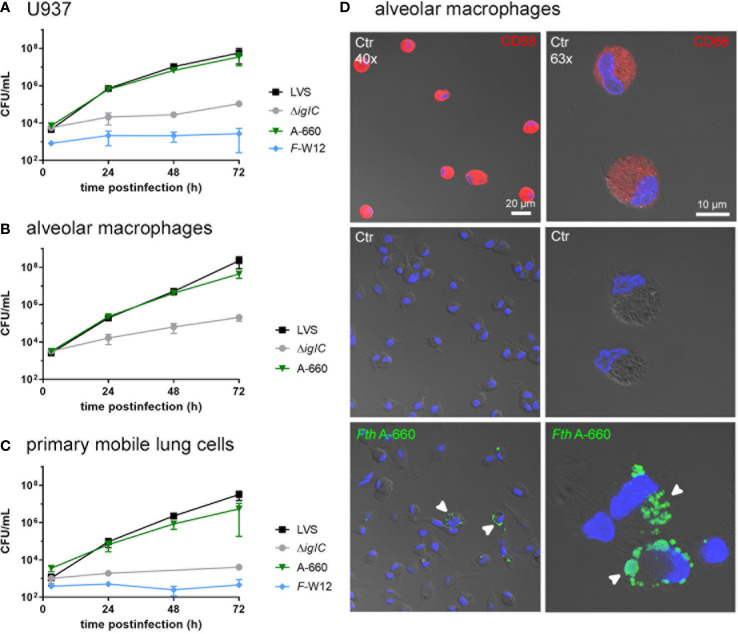
Infection of macrophage-like cell line U937 **(A)**, primarily isolated human alveolar macrophages **(B)** and primary mobile cells of human lung tissue **(C)** with *Francisella*. Cells were infected with *Fth* LVS, *Fth* LVS Δ*iglC*, *Fth* wild-type A-660 and *F*-W12 for 2 h (MOI = 10). Gentamicin (50 µg/mL for 1 h) was used to eliminate remaining extracellular bacteria. At various time points of infection, the CFU/mL was determined by lysing the cells and plating suspension onto agar plates. Means with standard deviation are shown. n = 3. **(D)** *Fth* wild-type A-660 (*Fth* A-660) replication in primary human alveolar macrophages (AM). AM, isolated from fresh human lung tissue, were cultivated for 2 days and infected with *Fth* A-660 (MOI = 10). After 2 hours bacterial suspension was removed and cells were treated with 50 µg/ml gentamicin for 1 h After 48 h of infection, AM were fixed and stained for macrophage specific cell marker CD68 (red), *Fth* A-660 (green), nuclei were counterstained with DAPI (blue) and cell structure was visualized with differential interference contrast. AM, positive for CD68 (upper panel), control, negative for *Fth* A-660 (middle panel) and 48 h infected (lower panel). White arrowheads point to *Fth* A-660. Scale bar represents 10 and 20 µm. Representative figures of three independent experiments are shown.

We next aimed to compare the results obtained by the *ex vivo* model with “classical” single cell type infection models using U937 macrophages and primarily isolated human alveolar macrophages as well as multicellular primarily isolated mobile human lung cells (**Figures 5A–C**). Cells were infected with *Fth* LVS, *Fth* LVS Δ*iglC*, *Fth* wild-type A-660 and *F*-W12 (MOI = 10) for 2 h and subsequently treated with gentamicin (50 µg/mL for 1 h) to eliminate remaining extracellular bacteria. After 3 h, 24 h, 48 h and 72 h of infection the CFU/mL was determined. Here, as observed in the human lung infection model, only *Fth* LVS and *Fth* A-660 were able to replicate within cells tested (**Figures 5A–C**). In U937 macrophages, both strains similarly replicated (**Figure 5A**), whereas in primary alveolar macrophages (**Figure 5B**) and primary mobile lung cells (**Figure 5C**), the *Fth* LVS strain showed higher growth rates compared to *Fth* A-660 (6-fold higher in alveolar macrophages and 19-fold in mobile lung cells, **Figures 5C**). Hence, these findings differ from the results obtained using the human lung *ex vivo* infection model, in which *Fth* A-660 showed a higher replication rate compared to *Fth* LVS (**Figure 2A**). However, *Fth* LVS Δ*iglC* and *F*-W12 did not significantly replicate in these cell culture models, except for an increase of CFU/mL observed for Δ*iglC* in infected primary alveolar macrophages after 72 h, indicating extracellular growth (64-fold induction, **Figure 3B**). Moreover, the intracellular localization of *Fth* A-660 in primarily isolated alveolar macrophages was confirmed by spectral immunofluorescence, as shown in **Figure 5D**.

### 3.4 Co-infection of U937 macrophages and human lung explants by *Fth* LVS and *Fth* A-660

**Figure 6:**
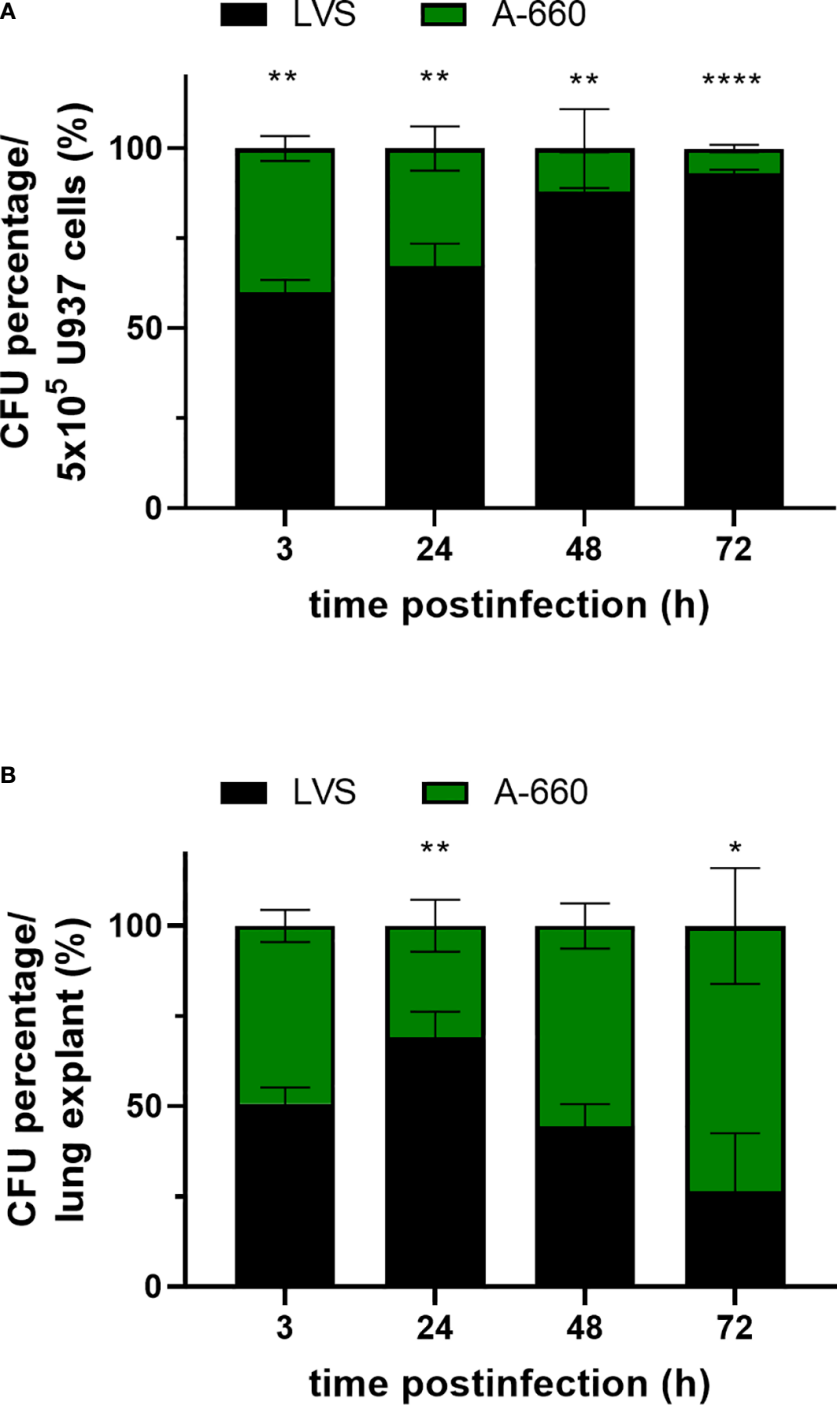
**(A)** Co-infection assay using U937 cells. Macrophages were competitively infected with *Fth* LVS and *Fth* A-660 (MOI = 10) for 2 h and treated afterwards with gentamicin (50 µg/mL for 1 h). At various time points, the CFU was determined by lysing U937 cells and plating onto MTKH agar plates partly supplemented with erythromycin to distinguish between *Fth* LVS (resistant) and *Fth* A-660 (sensitive). The percentage proportion of CFU per well (5 × 10^5^cells) is shown for *Fth* LVS (black) and *Fth* A-660 (green). **(B)** Co-infection assay using human lung tissue explants. Explants were competitively infected with *Fth* LVS and *Fth* A-660, as described in figure legend 1. The percentage of CFU per lung explant is shown for *Fth* LVS (black) and *Fth* A-660 (green). Means with standard deviation of three **(A)** and four **(B)** independent experiments, respectively, are shown and statistical analysis were performed using a two-tailed *t* test with *P < 0.05; **P < 0.001; ***P < 0.0001; ****P < 0.00001.

Having observed differences in intracellular growth of *Fth* LVS and *Fth* A-660 depending on the infection model used, *ex vivo* (lung explants) or *in vitro* (U937), we tested both models by co-infection assays. U937 cells were simultaneously infected with both strains at a ratio of 1:1 representing a MOI of 10 in total. Cells were infected for 2 h and subsequently treated with gentamicin for 1 h. After 3 h, 24 h, 48 h and 72 h of infection, the CFU/mL was determined by lysing cells and plating onto MTKH agar plates partly supplemented with erythromycin to distinguish the two strains. *Fth* LVS belongs to erythromycin-resistant biovar II, whereas *Fth* A-660 belongs to erythromycin-sensitive biovar I. In **Figure 6A**, the CFU percentage of *Fth* LVS and *Fth* A-660 per 5 × 10^5^ U937 cells is shown. After infection and gentamicin treatment (3 h), LVS represented 59.9% and A-660 40.1% of bacteria obtained intracellularly in U937 macrophages (p = 0.002). In the course of infection, the percentage of LVS continuously increased up to 93.2% after 72 h (p < 0.000001). Thus, LVS significantly outcompeted A-660 during a co-infection in U937 macrophages. In contrast, the A-660 wild-type strain outcompeted the LVS strain during a co-infection of human lung explants by both strains (**Figure 6B**). Human lung explants were equally infected with both strains after 3 h (LVS: 50.8%; A-660: 49.2%). After 24 h of co-infection, LVS represented a significantly higher percentage in the explants (69.1%, p = 0.0029), whereas the proportion of A-660 continuously increased up to 73.5% after 48 h and 72 h (72h: p = 0.0231). Hence, the attenuated phenotype (reduced virulence) of *Fth* LVS is only detectable in co-infections using human lung explants.

## 4 Discussion

In this study, we successfully adapted a human lung *ex vivo* infection model for analysis of *Francisella* strains, including *Fth* and an environmental *Francisella* strain. In addition to *Fth* LVS, we used *Fth* strain A-660 obtained from a patient suffering from pulmonic tularemia (Appelt et al., 2019). *Fth* LVS is an attenuated laboratory strain and often used to understand virulence and pathogenicity of *Francisella* (Fortier et al., 1991; Rick Lyons and Wu, 2007; Hall et al., 2008). In contrast, *Fth* A-660 represents a virulent wild-type strain occurring naturally and having caused tularemia in a patient. However, wild-type strains of *Fth* have rarely been examined in *Francisella* studies (Rick Lyons and Wu, 2007). In our *ex vivo* infection model, *Fth* LVS and six *Fth* wild-type strains replicated in human lung explants (**Figures 2A, B**). The observed replication is assumed to be intracellular, since the *Fth* LVS Δ*iglC* mutant strain, which has been shown to be unable to replicate intracellularly (Golovliov et al., 1997; Golovliov et al., 2003; Lindgren et al., 2004; Santic et al., 2005), did not multiply in the model (**Figure 2A**). An FPI-null mutant strain of *F. novicida* is able to grow extracellularly, when it is in contact with host cells supporting the conception that intracellular growth of *Fth* occurs in human lung explants (Rytter et al., 2021). We observed a slightly enhanced growth of A-660 in human lung explants compared to LVS (**Figure 2A**), but a similar replication of both strains in human U937 macrophages and primary alveolar macrophages (**Figures 4A–C**). In contrast, an *Fth* strain-specific virulence has been indicated by other studies using human monocytes (THP-1), murine macrophages (J774A.1; (Matz and Petrosino, 2021)) and a co-culture of human hepatocytes and macrophages (Rennert et al., 2016).These findings can, however, only be considered comparable with our results to a certain extent due to different cell types and experimental procedures used. To investigate *Francisella* virulence different models has been used so far, including animal models (see above and below), the Fruit Fly (*Drosophila melanogaster* (Vonkavaara et al., 2008; Ahlund et al., 2010; Asare et al., 2010), the larvae of the Greater Wax Moth (*Galleria mellonella*, (Aperis et al., 2007; Thelaus et al., 2018; Asai et al., 2023)) and *Dictyostelium discoideum* (Lampe et al., 2015; Brenz et al., 2018), as well as cell-dependent models such as *Drosophila* S2 cells (Santic et al., 2009; Asare et al., 2010) and macrophages (Qin and Mann, 2006; Maier et al., 2007; Rasmussen et al., 2015; Matz and Petrosino, 2021). However, it is always the question of transferability of results obtained by animal- or cell-line models to the human host.

The human lung *ex vivo* infection model clearly underpinned the higher virulence of the *Fth* wild-type (shown by a higher multiplication rate in human lung explants than LVS). Especially, when human lung explants were simultaneously challenged with both *Fth* strains, the wild-type A-660 outcompeted LVS demonstrating its higher virulence (**Figure 6**). Generally, the virulence of *Fth* depends on the route of infection and the bacterial dose; and is relatively low for humans when the pathogen is inhaled (Saslaw et al., 1961a; Saslaw et al., 1961b; Tigertt, 1962; Hall et al., 2008). After intranasal and aerosol uptake, 500 - 10^4^ *Fth* bacteria are needed for LD100 in mice and 10^8^ *Fth* LVS bacteria for a human infection (Saslaw et al., 1961a; Tigertt, 1962; Fortier et al., 1991; Duckett et al., 2005; Wu et al., 2005; Bosio et al., 2007). In our *ex vivo* model, the bacterial growth of *Francisella* increased up to 1.5-log levels which is lower compared to growth of other bacterial pathogens, such as e.g. pneumococci, which showed an increase of 3-log levels (Szymanski et al., 2012). Perspectivity, it might be interesting to determine the minimal doses for a successful growth in human lung explants and, thus, to determine the minimal infectious doses of diverse *Francisella* strains, particularly of highly virulent *Ftt*.

Using the human lung *ex vivo* infection model, we demonstrated that the environmental aquatic *Francisella* species *F*-W12 was not able to grow in the explants but could survive over three days similarly to *Fth* LVS Δ*iglC* strain. In fact, *F*-W12 does not exhibit the *Francisella* pathogenicity island encoding a type VI secretion system (Rydzewski et al., 2014). On the other hand, an alternative putative type VI secretion system has been identified *in silico* within its genome and several virulence factors has been found and experimentally confirmed (Köppen et al., 2019). However, this species does not seem to be as virulent as *Fth* and its putative pathogenicity for humans still needs to be verified.

In this study, we also aimed to identify the human lung cell types involved in human lung infections caused by *Francisella*. Spectral immunofluorescence and electron microscopy of *Fth* A-660 infected lung explants revealed that *Fth* A-660 was mainly detected in alveolar macrophages. Additionally, lymphocytes, granulocytes and fibrocytes have sporadically been infected by *Fth* (**Figures 3A–C**, **S3**). So far, only mouse (Bosio and Dow, 2005; Bosio et al., 2007; Forestal et al., 2007; Gentry et al., 2007; Hall et al., 2008; Mares et al., 2008; Yu et al., 2008) and primate infection models (Eigelsbach et al., 1962; White et al., 1964; Hall et al., 1973) have been used to identify the cell types involved in *Francisella* infection. Here, it was shown that the most infected cell types are alveolar macrophages, in particular at an early stage of infection (Hall et al., 2008). In mice, also other cell types are used for replication by *Francisella* over time, including dendritic cells and neutrophils, whereby the latter become the most-infected cell type in later stages of infection (Hall et al., 2008). The human lung *ex vivo* infection model does not allow an investigation of adaptive immune response and leukocyte recruitment. Therefore, further experiments are needed to study more closely intracellular replication niches of *Francisella* in dendritic cells or neutrophils. Furthermore, *Francisella* is able to infect human and mouse lung alveolar epithelial type II cells *in vitro* and *in vivo* (Gentry et al., 2007; Hall et al., 2007; Craven et al., 2008; Faron et al., 2015). Faron *et* al. hypothesized that after entry of *Francisella* into lung alveoli, bacteria infect and replicate either in alveolar macrophages or in alveolar epithelial type II cells (Faron et al., 2015). Both events lead to the epithelial barrier being crossed and thus, allow an interaction with surrounding cells, including endothelial cells. Consequently, *Francisella* bacteria are able to enter the lung blood capillaries and subsequently the blood stream. Although *Francisella* are not able to grow intracellularly in erythrocytes, the bacteria can use these to spread into the whole body (Horzempa et al., 2011; Schmitt et al., 2017; Cantlay et al., 2022). *Francisella* has also been shown to exist cell-free and extracellularly in blood and necrotic lesions in the lungs of infected mice (Bosio et al., 2007; Forestal et al., 2007; Yu et al., 2008). We also detected extracellular *Francisella* bacteria in the connective lung tissue. That partly supports the model of *Francisella* infection within the lung by Faron et al., although we did neither find infected alveolar epithelial type II cells nor endothelial cells in the investigated lung explants so far.

We also assessed how *Francisella* activated the host immune response in *ex vivo* infected human lung tissue. Cytokine and chemokine levels in lung explants from different donors and even from the same donor varied more or less. Moreover, variations were not only observed in supernatants of infected explants, but also of non-infected control explants (see **Figure S2**). Another variation has been shown in a study investigating cytokine response of naturally acquired tularemia among a group of nine hare hunters (Jacob et al., 2020). Although cytokine response (IL-8, IP-10, MCP-1 and MIP-1α) showed considerable changes during the acute phase of infection, this remained statistically insignificant. Among the tendencies in cytokine response revealed by cytokine measurements conducted in our study, it could be demonstrated that secretion of IL-1β, IL-6, MCP-1 and IP-10, known as proinflammatory stimulators produced by macrophages, seemed to be slightly lower in explants infected by *Fth* A-660 compared to those infected by *Fth* LVS (see **Figure 4**). Our data are consistent with other studies showing an active suppression of the immune response and cytokine release of virulent *Francisella* strains compared to non- or less-virulent strains in the early phase of infection (Bosio and Dow, 2005; Mares et al., 2008; Gillette et al., 2014). In particular, the upregulation of proinflammatory TNF-α is delayed by an active suppression after *Francisella* infection. This *Francisella*-induced active immune repression might explain the generally low levels of cytokine in supernatants of *Francisella* infected lung explants in our model compared to published data based on diverse pathogens, such as e.g. *S. pneumoniae* (e.g. TNF-α: max. 30000 pg/g lung tissue; IL-1β: max. of 40000 pg/g lung tissue; IL-8: max. of 5000 ng/g tissue; IL-6: max. of 3000 ng/g tissue (Szymanski et al., 2012; Fatykhova et al., 2015; Berg et al., 2017)). Importantly, the immune response suppression was not observed for the environmental strain *F*-W12 (**Figures 4**, **S2**). Here, we detected cytokine levels that rose up to 40 times higher.

## 5 Conclusion

For the first time, we successfully implemented an *ex vivo* infection model using human lung tissue for *Francisella*. In contrast to a 2D single cell type infection model, this model mimics the 3D *in vivo* situation with diverse cell types responding to *Francisella* on a small scale. This enabled us to detect the specific ability of wild-type A-660 to replicate within the cells and tissue, as well as to manipulate the immune response. Thus, the human lung *ex vivo* model is apt to be used to discriminate virulent from less- or non-virulent *Francisella* species and to investigate the role of specific virulence factors.

## Data availability statement

The original contributions presented in the study are included in the article/**Supplementary Material**. Further inquiries can be directed to the corresponding authors.

## Ethics statement

The studies involving human participants were reviewed and approved by Ethics committee of the Charité - Universitätsmedizin Berlin, Germany (project EA2/079/13). The patients/participants provided their written informed consent to participate in this study.

## Author contributions

KH and KK provided the expertise in the field of *Francisella*. DF, SH and AH provided the expertise in the field of human lung *ex vivo* infection models. Theoretical and practical advices were given by KH, KK, DF, SH and AH. KH and SH coordinated and supervised the present work. Human lung explants were provided by MG and DF. KK, DF, HG, JR and KR performed the experiments and analyzed the data. KK and KH drafted the manuscript. DF, GH, JR, DT, AH and SH revised the manuscript critically. All authors contributed to the article and approved the submitted version.

## References

[B1] Ahlund M. K. Rydén P. Sjöstedt A. Stöven S. (2010). Directed screen of francisella novicida virulence determinants using drosophila melanogaster. Infect. Immun. 78, 3118–3128. doi: 10.1128/IAI.00146-10 20479082 PMC2897386

[B2] Anthony L. D. Burke R. D. Nano F. E. (1991). Growth of francisella spp. in rodent macrophages. Infect. Immun. 59, 3291–3296. doi: 10.1128/iai.59.9.3291-3296.1991 1879943 PMC258167

[B3] Aperis G. Fuchs B. B. Anderson C. A. Warner J. E. Calderwood S. B. Mylonakis E. (2007). Galleria mellonella as a model host to study infection by the francisella tularensis live vaccine strain. Microbes Infect. 9, 729–734. doi: 10.1016/j.micinf.2007.02.016 17400503 PMC1974785

[B4] Appelt S. Köppen K. Radonic A. Drechsel O. Jacob D. Grunow R.. (2019). Genetic diversity and spatial segregation of francisella tularensis subspecies holarctica in Germany. Front. Cell Infect. Microbiol. 9, 376. doi: 10.3389/fcimb.2019.00376 31781515 PMC6851236

[B5] Asai M. Li Y. Newton S. M. Robertson B. D. Langford P. R. (2023). Galleria mellonella-intracellular bacteria pathogen infection models: the ins and outs. FEMS Microbiol. Rev. 47, 1–32. doi: 10.1093/femsre/fuad011 PMC10045907 36906279

[B6] Asare R. Akimana C. Jones S. Abu Kwaik Y. (2010). Molecular bases of proliferation of francisella tularensis in arthropod vectors. Environ. Microbiol. 12, 2587–2612. doi: 10.1111/j.1462-2920.2010.02230.x 20482589 PMC2957557

[B7] Bandouchova H. Sedlackova J. Pohanka M. Novotny L. Hubalek M. Treml F.. (2009). Tularemia induces different biochemical responses in BALB/c mice and common voles. BMC Infect. Dis. 9, 101. doi: 10.1186/1471-2334-9-101 19558687 PMC2711074

[B8] Becker S. Lochau P. Jacob D. Heuner K. Grunow R. (2016). Successful re-evaluation of broth medium T for growth of francisella tularensis ssp. and other highly pathogenic bacteria. J. Microbiol. Methods 121, 5–7. doi: 10.1016/j.mimet.2015.11.018 26658853

[B9] Ben Nasr A. Haithcoat J. Masterson J. E. Gunn J. S. Eaves-Pyles T. Klimpel G. R. (2006). Critical role for serum opsonins and complement receptors CR3 (CD11b/CD18) and CR4 (CD11c/CD18) in phagocytosis of francisella tularensis by human dendritic cells (DC): uptake of francisella leads to activation of immature DC and intracellular survival of the bacteria. J. Leukoc. Biol. 80, 774–786. doi: 10.1189/jlb.1205755 16857732

[B10] Berg J. Zscheppang K. Fatykhova D. Tonnies M. Bauer T. T. Schneider P.. (2017). Tyk2 as a target for immune regulation in human viral/bacterial pneumonia. Eur. Respir. J. 50. doi: 10.1183/13993003.01953-2016 28705941

[B11] Birdsell D. N. Stewart T. Vogler A. J. Lawaczeck E. Diggs A. Sylvester T. L.. (2009). Francisella tularensis subsp. novicida isolated from a human in Arizona. BMC Res. Notes 2, 223. doi: 10.1186/1756-0500-2-223 19895698 PMC2780447

[B12] Booth J. L. Duggan E. S. Patel V. I. Langer M. Wu W. Braun A.. (2016). Bacillus anthracis spore movement does not require a carrier cell and is not affected by lethal toxin in human lung models. Microbes Infect. 18, 615–626. doi: 10.1016/j.micinf.2016.06.004 27320392 PMC5534360

[B13] Bosio C. M. Bielefeldt-Ohmann H. Belisle J. T. (2007). Active suppression of the pulmonary immune response by francisella tularensis Schu4. J. Immunol. 178, 4538–4547. doi: 10.4049/jimmunol.178.7.4538 17372012

[B14] Bosio C. M. Dow S. W. (2005). Francisella tularensis induces aberrant activation of pulmonary dendritic cells. J. Immunol. 175, 6792–6801. doi: 10.4049/jimmunol.175.10.6792 16272336

[B15] Brenz Y. Winther-Larsen H. C. Hagedorn M. (2018). Expanding francisella models: pairing up the soil amoeba dictyostelium with aquatic francisella. Int. J. Med. Microbiol. 308, 32–40. doi: 10.1016/j.ijmm.2017.08.001 28843671

[B16] Brett M. E. Respicio-Kingry L. B. Yendell S. Ratard R. Hand J. Balsamo G.. (2014). Outbreak of francisella novicida bacteremia among inmates at a louisiana correctional facility. Clin. Infect. Dis. 59, 826–833. doi: 10.1093/cid/ciu430 24944231

[B17] Bröms J. E. Sjöstedt A. Lavander M. (2010). The role of the francisella tularensis pathogenicity island in type VI secretion, intracellular survival, and modulation of host cell signaling. Front. Microbiol. 1, 136. doi: 10.3389/fmicb.2010.00136 21687753 PMC3109350

[B18] Cantlay S. Kaftanic C. Horzempa J. (2022). PdpC, a secreted effector protein of the type six secretion system, is required for erythrocyte invasion by francisella tularensis LVS. Front. Cell Infect. Microbiol. 12, 979693. doi: 10.3389/fcimb.2022.979693 36237421 PMC9552824

[B19] Clemens D. L. Lee B. Y. Horwitz M. A. (2004). Virulent and avirulent strains of francisella tularensis prevent acidification and maturation of their phagosomes and escape into the cytoplasm in human macrophages. Infect. Immun. 72, 3204–3217. doi: 10.1128/IAI.72.6.3204-3217.2004 15155622 PMC415696

[B20] Clemens D. L. Lee B. Y. Horwitz M. A. (2018). The francisella type VI secretion system. Front. Cell Infect. Microbiol. 8, 121. doi: 10.3389/fcimb.2018.00121 29740542 PMC5924787

[B21] Craven R. R. Hall J. D. Fuller J. R. Taft-Benz S. Kawula T. H. (2008). Francisella tularensis invasion of lung epithelial cells. Infect. Immun. 76, 2833–2842. doi: 10.1128/IAI.00043-08 18426871 PMC2446690

[B22] Dennis D. T. Inglesby T. V. Henderson D. A. Bartlett J. G. Ascher M. S. Eitzen E.. (2001). Tularemia as a biological weapon: medical and public health management. JAMA 285, 2763–2773. doi: 10.1001/jama.285.21.2763 11386933

[B23] Duckett N. S. Olmos S. Durrant D. M. Metzger D. W. (2005). Intranasal interleukin-12 treatment for protection against respiratory infection with the francisella tularensis live vaccine strain. Infect. Immun. 73, 2306–2311. doi: 10.1128/IAI.73.4.2306-2311.2005 15784575 PMC1087453

[B24] Eigelsbach H. T. Tulis J. J. Mcgavran M. H. White J. D. (1962). LIVE TULAREMIA VACCINE i. : host-parasite relationship in monkeys vaccinated intracutaneously or aerogenically. J. Bacteriol. 84, 1020–1027. doi: 10.1128/jb.84.5.1020-1027.1962 16561967 PMC278004

[B25] Ellis J. Oyston P. C. Green M. Titball R. W. (2002). Tularemia. Clin. Microbiol. Rev. 15, 631–646. doi: 10.1128/CMR.15.4.631-646.2002 12364373 PMC126859

[B26] Escudero R. Elia M. Saez-Nieto J. A. Menendez V. Toledo A. Royo G.. (2010). A possible novel francisella genomic species isolated from blood and urine of a patient with severe illness. Clin. Microbiol. Infect. 16, 1026–1030. doi: 10.1111/j.1469-0691.2009.03029.x 19709068

[B27] Faron M. Fletcher J. R. Rasmussen J. A. Apicella M. A. Jones B. D. (2015). Interactions of francisella tularensis with alveolar type II epithelial cells and the murine respiratory epithelium. PloS One 10, e0127458. doi: 10.1371/journal.pone.0127458 26010977 PMC4444194

[B28] Fatykhova D. Rabes A. Machnik C. Guruprasad K. Pache F. Berg J.. (2015). Serotype 1 and 8 pneumococci evade sensing by inflammasomes in human lung tissue. PloS One 10, e0137108. doi: 10.1371/journal.pone.0137108 26317436 PMC4552725

[B29] Foley J. E. Nieto N. C. (2010). Tularemia. Vet. Microbiol. 140, 332–338. doi: 10.1016/j.vetmic.2009.07.017 19713053

[B30] Forestal C. A. Malik M. Catlett S. V. Savitt A. G. Benach J. L. Sellati T. J.. (2007). Francisella tularensis has a significant extracellular phase in infected mice. J. Infect. Dis. 196, 134–137. doi: 10.1086/518611 17538893

[B31] Fortier A. H. Slayter M. V. Ziemba R. Meltzer M. S. Nacy C. A. (1991). Live vaccine strain of francisella tularensis: infection and immunity in mice. Infect. Immun. 59, 2922–2928. doi: 10.1128/iai.59.9.2922-2928.1991 1879918 PMC258114

[B32] Froböse N. J. Masjosthusmann K. Huss S. Correa-Martinez C. L. Mellmann A. Schuler F.. (2020). A child with soft-tissue infection and lymphadenitis. New Microbes New Infect. 38, 100819. doi: 10.1016/j.nmni.2020.100819 33304596 PMC7718473

[B33] Ganbat D. Seehase S. Richter E. Vollmer E. Reiling N. Fellenberg K.. (2016). Mycobacteria infect different cell types in the human lung and cause species dependent cellular changes in infected cells. BMC Pulm. Med. 16, 19. doi: 10.1186/s12890-016-0185-5 26803467 PMC4724406

[B34] Gentry M. Taormina J. Pyles R. B. Yeager L. Kirtley M. Popov V. L.. (2007). Role of primary human alveolar epithelial cells in host defense against francisella tularensis infection. Infect. Immun. 75, 3969–3978. doi: 10.1128/IAI.00157-07 17502386 PMC1951971

[B35] Gillette D. D. Curry H. M. Cremer T. Ravneberg D. Fatehchand K. Shah P. A.. (2014). Virulent type a francisella tularensis actively suppresses cytokine responses in human monocytes. Front. Cell Infect. Microbiol. 4, 45. doi: 10.3389/fcimb.2014.00045 24783062 PMC3988375

[B36] Glynn A. R. Alves D. A. Frick O. Erwin-Cohen R. Porter A. Norris S.. (2015). Comparison of experimental respiratory tularemia in three nonhuman primate species. Comp. Immunol. Microbiol. Infect. Dis. 39, 13–24. doi: 10.1016/j.cimid.2015.01.003 25766142 PMC4397973

[B37] Golovliov I. Ericsson M. Sandstrom G. Tarnvik A. Sjostedt A. (1997). Identification of proteins of francisella tularensis induced during growth in macrophages and cloning of the gene encoding a prominently induced 23-kilodalton protein. Infect. Immun. 65, 2183–2189. doi: 10.1128/iai.65.6.2183-2189.1997 9169749 PMC175301

[B38] Golovliov I. Sjostedt A. Mokrievich A. Pavlov V. (2003). A method for allelic replacement in francisella tularensis. FEMS Microbiol. Lett. 222, 273–280. doi: 10.1016/S0378-1097(03)00313-6 12770718

[B39] Graham J. G. Winchell C. G. Kurten R. C. Voth D. E. (2016). Development of an ex vivo tissue platform to study the human lung response to coxiella burnetii. Infect. Immun. 84, 1438–1445. doi: 10.1128/IAI.00012-16 26902725 PMC4862715

[B40] Grund C. Hoffmann D. Ulrich R. Naguib M. Schinkothe J. Hoffmann B.. (2018). A novel European H5N8 influenza a virus has increased virulence in ducks but low zoonotic potential. Emerg. Microbes Infect. 7, 132. doi: 10.1038/s41426-018-0130-1 30026505 PMC6053424

[B41] Grunow R. Splettstoesser W. Mcdonald S. Otterbein C. O'brien T. Morgan C.. (2000). Detection of francisella tularensis in biological specimens using a capture enzyme-linked immunosorbent assay, an immunochromatographic handheld assay, and a PCR. Clin. Diagn. Lab. Immunol. 7, 86–90. doi: 10.1128/CDLI.7.1.86-90.2000 10618283 PMC95828

[B42] Hall J. D. Craven R. R. Fuller J. R. Pickles R. J. Kawula T. H. (2007). Francisella tularensis replicates within alveolar type II epithelial cells *in vitro* and *in vivo* following inhalation. Infect. Immun. 75, 1034–1039. doi: 10.1128/IAI.01254-06 17088343 PMC1828526

[B43] Hall W. C. Kovatch R. M. Schricker R. L. (1973). Tularaemic pneumonia: pathogenesis of the aerosol-induced disease in monkeys. J. Pathol. 110, 193–201. doi: 10.1002/path.1711100302 4200656

[B44] Hall J. D. Woolard M. D. Gunn B. M. Craven R. R. Taft-Benz S. Frelinger J. A.. (2008). Infected-host-cell repertoire and cellular response in the lung following inhalation of francisella tularensis schu S4, LVS, or U112. Infect. Immun. 76, 5843–5852. doi: 10.1128/IAI.01176-08 18852251 PMC2583552

[B45] Hennebique A. Caspar Y. Maurin M. Boisset S. Pelloux I. Gallego-Hernanz M. P.. (2022). Ulceroglandular infection and bacteremia caused by francisella salimarina in immunocompromised patient, France. Emerg. Infect. Dis. 28, 465–467. doi: 10.3201/eid2802.211380 35076000 PMC8798692

[B46] Hocke A. C. Becher A. Knepper J. Peter A. Holland G. Tonnies M.. (2013). Emerging human middle East respiratory syndrome coronavirus causes widespread infection and alveolar damage in human lungs. Am. J. Respir. Crit. Care Med. 188, 882–886. doi: 10.1164/rccm.201305-0954LE 24083868

[B47] Hollis D. G. Weaver R. E. Steigerwalt A. G. Wenger J. D. Moss C. W. Brenner D. J. (1989). Francisella philomiragia comb. nov. (formerly yersinia philomiragia) and francisella tularensis biogroup novicida (formerly francisella novicida) associated with human disease. J. Clin. Microbiol. 27, 1601–1608. doi: 10.1128/jcm.27.7.1601-1608.1989 2671019 PMC267622

[B48] Hönzke K. Obermayer B. Mache C. Fathykova D. Kessler M. Dokel S.. (2022). Human lungs show limited permissiveness for SARS-CoV-2 due to scarce ACE2 levels but virus-induced expansion of inflammatory macrophages. Eur. Respir. J. 60, 210275. doi: 10.1183/13993003.02725-2021 PMC9712848 35728978

[B49] Horzempa J. O'dee D. M. Shanks R. M. Nau G. J. (2010). Francisella tularensis DeltapyrF mutants show that replication in nonmacrophages is sufficient for pathogenesis *in vivo* . Infect. Immun. 78, 2607–2619. doi: 10.1128/IAI.00134-10 20385757 PMC2876533

[B50] Horzempa J. O'dee D. M. Stolz D. B. Franks J. M. Clay D. Nau G. J. (2011). Invasion of erythrocytes by francisella tularensis. J. Infect. Dis. 204, 51–59. doi: 10.1093/infdis/jir221 21628658 PMC3105038

[B51] Jacob D. Barduhn A. Tappe D. Rauch J. Heuner K. Hierhammer D.. (2020). Outbreak of tularemia in a group of hunters in Germany in 2018-kinetics of antibody and cytokine responses. Microorganisms 8, 1645. doi: 10.3390/microorganisms8111645 33114188 PMC7690809

[B52] Jäger J. Marwitz S. Tiefenau J. Rasch J. Shevchuk O. Kugler C.. (2014). Human lung tissue explants reveal novel interactions during legionella pneumophila infections. Infect. Immun. 82, 275–285. doi: 10.1128/IAI.00703-13 24166955 PMC3911869

[B53] Knepper J. Schierhorn K. L. Becher A. Budt M. Tonnies M. Bauer T. T.. (2013). The novel human influenza A(H7N9) virus is naturally adapted to efficient growth in human lung tissue. mBio 4, e00601–e00613. doi: 10.1128/mBio.00601-13 24105764 PMC3791893

[B54] Köppen K. Chen F. Rydzewski K. Einenkel R. Bottcher T. Morguet C.. (2019). Screen for fitness and virulence factors of francisella sp. strain W12-1067 using amoebae. Int. J. Med. Microbiol. 309, 151341. doi: 10.1016/j.ijmm.2019.151341 31451389

[B55] Kostiala A. A. Mcgregor D. D. Logie P. S. (1975). Tularaemia in the rat. i. the cellular basis on host resistance to infection. Immunology 28, 855–869. 236983 PMC1445928

[B56] Kreitmann L. Terriou L. Launay D. Caspar Y. Courcol R. Maurin M.. (2015). Disseminated infection caused by francisella philomiragia, France 2014. Emerg. Infect. Dis. 21, 2260–2261. doi: 10.3201/eid2112.150615 26583375 PMC4672438

[B57] Lampe E. O. Brenz Y. Herrmann L. Repnik U. Griffiths G. Zingmark C.. (2015). Dissection of francisella-host cell interactions in dictyostelium discoideum. Appl. Environ. Microbiol. 82, 1586–1598. doi: 10.1128/AEM.02950-15 26712555 PMC4771330

[B58] Lindgren H. Golovliov I. Baranov V. Ernst R. K. Telepnev M. Sjostedt A. (2004). Factors affecting the escape of francisella tularensis from the phagolysosome. J. Med. Microbiol. 53, 953–958. doi: 10.1099/jmm.0.45685-0 15358816

[B59] Maier T. M. Casey M. S. Becker R. H. Dorsey C. W. Glass E. M. Maltsev N.. (2007). Identification of francisella tularensis Himar1-based transposon mutants defective for replication in macrophages. Infect. Immun. 75, 5376–5389. doi: 10.1128/IAI.00238-07 17682043 PMC2168294

[B60] Mailman T. L. Schmidt M. H. (2005). Francisella philomiragia adenitis and pulmonary nodules in a child with chronic granulomatous disease. Can. J. Infect. Dis. Med. Microbiol. 16, 245–248. doi: 10.1155/2005/486417 18159552 PMC2095034

[B61] Mares C. A. Ojeda S. S. Morris E. G. Li Q. Teale J. M. (2008). Initial delay in the immune response to francisella tularensis is followed by hypercytokinemia characteristic of severe sepsis and correlating with upregulation and release of damage-associated molecular patterns. Infect. Immun. 76, 3001–3010. doi: 10.1128/IAI.00215-08 18411294 PMC2446713

[B62] Matz L. M. Petrosino J. F. (2021). A study of innate immune kinetics reveals a role for a chloride transporter in a virulent francisella tularensis type b strain. Microbiologyopen 10, e1170. doi: 10.1002/mbo3.1170 33970545 PMC8483402

[B63] Maurin M. (2015). Francisella tularensis as a potential agent of bioterrorism? Expert Rev. Anti Infect. Ther. 13, 141–144. doi: 10.1586/14787210.2015.986463 25413334

[B64] Maurin M. Gyuranecz M. (2016). Tularaemia: clinical aspects in Europe. Lancet Infect. Dis. 16, 113–124. doi: 10.1016/S1473-3099(15)00355-2 26738841

[B65] Mccrumb F. R. (1961). Aerosol infection of man with pasteurella tularensis. Bacteriol. Rev. 25, 262–267. doi: 10.1128/br.25.3.262-267.1961 16350172 PMC441102

[B66] Melillo A. Sledjeski D. D. Lipski S. Wooten R. M. Basrur V. Lafontaine E. R. (2006). Identification of a francisella tularensis LVS outer membrane protein that confers adherence to A549 human lung cells. FEMS Microbiol. Lett. 263, 102–108. doi: 10.1111/j.1574-6968.2006.00413.x 16958857

[B67] Moreau G. B. Mann B. J. (2013). Adherence and uptake of francisella into host cells. Virulence 4, 826–832. doi: 10.4161/viru.25629 23921460 PMC3925714

[B68] Nelson M. Lever M. S. Dean R. E. Savage V. L. Salguero F. J. Pearce P. C.. (2010). Characterization of lethal inhalational infection with francisella tularensis in the common marmoset (Callithrix jacchus). J. Med. Microbiol. 59, 1107–1113. doi: 10.1099/jmm.0.020669-0 20558585 PMC3052436

[B69] Owen C. R. Buker E. O. Jellison W. L. Lackman D. B. Bell J. F. (1964). Comparative studies of francisella tularensis and francisella novicida. J. Bacteriol. 87, 676–683. doi: 10.1128/jb.87.3.676-683.1964 14127585 PMC277070

[B70] Pavlovich N. V. Mishan'kin B. N. (1987). [Transparent nutrient medium for culturing francisella tularensis]. Antibiot. Med. Biotekhnol. 32, 133–137. 3551821

[B71] Peter A. Fatykhova D. Kershaw O. Gruber A. D. Rueckert J. Neudecker J.. (2017). Localization and pneumococcal alteration of junction proteins in the human alveolar-capillary compartment. Histochem. Cell Biol. 147, 707–719. doi: 10.1007/s00418-017-1551-y 28247028

[B72] Qin A. Mann B. J. (2006). Identification of transposon insertion mutants of francisella tularensis tularensis strain schu S4 deficient in intracellular replication in the hepatic cell line HepG2. BMC Microbiol. 6, 69. doi: 10.1186/1471-2180-6-69 16879747 PMC1557513

[B73] Rasmussen J. A. Fletcher J. R. Long M. E. Allen L. A. Jones B. D. (2015). Characterization of francisella tularensis schu S4 mutants identified from a transposon library screened for O-antigen and capsule deficiencies. Front. Microbiol. 6, 338. doi: 10.3389/fmicb.2015.00338 25999917 PMC4419852

[B74] Ray H. J. Chu P. Wu T. H. Lyons C. R. Murthy A. K. Guentzel M. N.. (2010). The Fischer 344 rat reflects human susceptibility to francisella pulmonary challenge and provides a new platform for virulence and protection studies. PloS One 5, e9952. doi: 10.1371/journal.pone.0009952 20376351 PMC2848594

[B75] Rennert K. Otto P. Funke H. Huber O. Tomaso H. Mosig A. S. (2016). A human macrophage-hepatocyte co-culture model for comparative studies of infection and replication of francisella tularensis LVS strain and subspecies holarctica and mediasiatica. BMC Microbiol. 16, 2. doi: 10.1186/s12866-015-0621-3 26739172 PMC4704405

[B76] Rick Lyons C. Wu T. H. (2007). Animal models of francisella tularensis infection. Ann. N. Y. Acad. Sci. 1105, 238–265. doi: 10.1196/annals.1409.003 17395735

[B77] Roberts L. M. Powell D. A. Frelinger J. A. (2018). Adaptive immunity to francisella tularensis and considerations for vaccine development. Front. Cell Infect. Microbiol. 8, 115. doi: 10.3389/fcimb.2018.00115 29682484 PMC5898179

[B78] Rydzewski K. Schulz T. Brzuszkiewicz E. Holland G. Lück C. Fleischer J.. (2014). Genome sequence and phenotypic analysis of a first German francisella sp. isolate (W12-1067) not belonging to the species francisella tularensis. BMC Microbiol. 14, 169. doi: 10.1186/1471-2180-14-169 24961323 PMC4230796

[B79] Rytter H. Jamet A. Ziveri J. Ramond E. Coureuil M. Lagouge-Roussey P.. (2021). The pentose phosphate pathway constitutes a major metabolic hub in pathogenic francisella. PloS Pathog. 17, e1009326. doi: 10.1371/journal.ppat.1009326 34339477 PMC8360588

[B80] Santic M. Akimana C. Asare R. Kouokam J. C. Atay S. Kwaik Y. A. (2009). Intracellular fate of francisella tularensis within arthropod-derived cells. Environ. Microbiol. 11, 1473–1481. doi: 10.1111/j.1462-2920.2009.01875.x 19220402

[B81] Santic M. Al-Khodor S. Abu Kwaik Y. (2010). Cell biology and molecular ecology of francisella tularensis. Cell Microbiol. 12, 129–139. doi: 10.1111/j.1462-5822.2009.01400.x 19863554

[B82] Santic M. Molmeret M. Klose K. E. Jones S. Kwaik Y. A. (2005). The francisella tularensis pathogenicity island protein IglC and its regulator MglA are essential for modulating phagosome biogenesis and subsequent bacterial escape into the cytoplasm. Cell Microbiol. 7, 969–979. doi: 10.1111/j.1462-5822.2005.00526.x 15953029

[B83] Saslaw S. Eigelsbach H. T. Prior J. A. Wilson H. E. Carhart S. (1961a). Tularemia vaccine study. II. respiratory challenge. Arch. Intern. Med. 107, 702–714. doi: 10.1001/archinte.1961.03620050068007 13746667

[B84] Saslaw S. Eigelsbach H. T. Wilson H. E. Prior J. A. Carhart S. (1961b). Tularemia vaccine study. i. intracutaneous challenge. Arch. Intern. Med. 107, 689–701. doi: 10.1001/archinte.1961.03620050055006 13746668

[B85] Schmitt D. M. Barnes R. Rogerson T. Haught A. Mazzella L. K. Ford M.. (2017). The role and mechanism of erythrocyte invasion by francisella tularensis. Front. Cell Infect. Microbiol. 7, 173. doi: 10.3389/fcimb.2017.00173 28536678 PMC5423315

[B86] Schulze C. Heuner K. Myrtennäs K. Karlsson E. Jacob D. Kutzer P.. (2016). High and novel genetic diversity of francisella tularensis in Germany and indication of environmental persistence. Epidemiol. Infect. 144, 3025–3036. doi: 10.1017/S0950268816001175 27356883 PMC9150394

[B87] Schwartz J. T. Barker J. H. Long M. E. Kaufman J. Mccracken J. Allen L. A. (2012). Natural IgM mediates complement-dependent uptake of francisella tularensis by human neutrophils via complement receptors 1 and 3 in nonimmune serum. J. Immunol. 189, 3064–3077. doi: 10.4049/jimmunol.1200816 22888138 PMC3436988

[B88] Staples J. E. Kubota K. A. Chalcraft L. G. Mead P. S. Petersen J. M. (2006). Epidemiologic and molecular analysis of human tularemia, united states 1964-2004. Emerg. Infect. Dis. 12, 1113–1118. doi: 10.3201/eid1207.051504 16836829 PMC3291054

[B89] Szymanski K. V. Toennies M. Becher A. Fatykhova D. N'guessan P. D. Gutbier B.. (2012). Streptococcus pneumoniae-induced regulation of cyclooxygenase-2 in human lung tissue. Eur. Respir. J. 40, 1458–1467. doi: 10.1183/09031936.00186911 22441740

[B90] Thelaus J. Lundmark E. Lindgren P. Sjodin A. Forsman M. (2018). Galleria mellonella reveals niche differences between highly pathogenic and closely related strains of francisella spp. Front. Cell Infect. Microbiol. 8, 188. doi: 10.3389/fcimb.2018.00188 29922601 PMC5996057

[B91] Tigertt W. D. (1962). Soviet viable pasteurella tularensis vaccines. a review of selected articles. Bacteriol. Rev. 26, 354–373. doi: 10.1128/br.26.3.354-373.1962 13985026 PMC441156

[B92] Tlapak H. Köppen K. Rydzewski K. Grunow R. Heuner K. (2018). Construction of a new phage integration vector pFIV-Val for use in different francisella species. Front. Cell Infect. Microbiol. 8, 75. doi: 10.3389/fcimb.2018.00075 29594068 PMC5861138

[B93] Twenhafel N. A. Alves D. A. Purcell B. K. (2009). Pathology of inhalational francisella tularensis spp. tularensis SCHU S4 infection in African green monkeys (Chlorocebus aethiops). Vet. Pathol. 46, 698–706. doi: 10.1354/vp.08-VP-0302-T-AM 19276059

[B94] Vonkavaara M. Telepnev M. V. Ryden P. Sjostedt A. Stoven S. (2008). Drosophila melanogaster as a model for elucidating the pathogenicity of francisella tularensis. Cell Microbiol. 10, 1327–1338. doi: 10.1111/j.1462-5822.2008.01129.x 18248629

[B95] Vuorte J. Jansson S. E. Repo H. (2001). Evaluation of red blood cell lysing solutions in the study of neutrophil oxidative burst by the DCFH assay. Cytometry 43, 290–296. doi: 10.1002/1097-0320(20010401)43:4<290::AID-CYTO1061>3.0.CO;2-X 11260596

[B96] Wagner C. Goldmann T. Rohmann K. Rupp J. Marwitz S. Rotta Detto Loria J.. (2015). Budesonide inhibits intracellular infection with non-typeable haemophilus influenzae despite its anti-inflammatory effects in respiratory cells and human lung tissue: a role for p38 MAP kinase. Respiration 90, 416–425. doi: 10.1159/000439226 26452008

[B97] Weinheimer V. K. Becher A. Tonnies M. Holland G. Knepper J. Bauer T. T.. (2012). Influenza a viruses target type II pneumocytes in the human lung. J. Infect. Dis. 206, 1685–1694. doi: 10.1093/infdis/jis455 22829640 PMC7107318

[B98] Wenger J. D. Hollis D. G. Weaver R. E. Baker C. N. Brown G. R. Brenner D. J.. (1989). Infection caused by francisella philomiragia (formerly yersinia philomiragia). a newly recognized human pathogen. Ann. Intern. Med. 110, 888–892. doi: 10.7326/0003-4819-110-11-888 2541646

[B99] Whipp M. J. Davis J. M. Lum G. De Boer J. Zhou Y. Bearden S. W.. (2003). Characterization of a novicida-like subspecies of francisella tularensis isolated in Australia. J. Med. Microbiol. 52, 839–842. doi: 10.1099/jmm.0.05245-0 12909664

[B100] White J. D. Rooney J. R. Prickett P. A. Derrenbacher E. B. Beard C. W. Griffith W. R. (1964). Pathogenesis of experimental respiratory tularemia in monkeys. J. Infect. Dis. 114, 277–283. doi: 10.1093/infdis/114.3.277 14183401

[B101] Wu T. H. Hutt J. A. Garrison K. A. Berliba L. S. Zhou Y. Lyons C. R. (2005). Intranasal vaccination induces protective immunity against intranasal infection with virulent francisella tularensis biovar a. Infect. Immun. 73, 2644–2654. doi: 10.1128/IAI.73.5.2644-2654.2005 15845466 PMC1087315

[B102] Yu J. J. Raulie E. K. Murthy A. K. Guentzel M. N. Klose K. E. Arulanandam B. P. (2008). The presence of infectious extracellular francisella tularensis subsp. novicida in murine plasma after pulmonary challenge. Eur. J. Clin. Microbiol. Infect. Dis. 27, 323–325. doi: 10.1007/s10096-007-0434-x 18087734

