# Supplementary material for: Ex vivo infection model for Francisella using human lung tissue
Source: Front Cell Infect Microbiol. 2023 Jul 10;13:1224356. doi: 10.3389/fcimb.2023.1224356 (PMC10365108; doi:10.3389/fcimb.2023.1224356)
Supplement: Supplementary file 1 [file DataSheet_1.docx]

Supplementary Material

*Ex vivo* infection model for *Francisella* using human lung tissue

**Kristin Köppen^1^** †**, Diana Fatykhova^2^** †**, Gudrun Holland^3^, Jessica Rauch^4^, Dennis Tappe^4^, Mareike Graff^5^, Kerstin Rydzewski^1^, Andreas C. Hocke^2^, Stefan Hippenstiel^2*^, Klaus Heuner^1^***

^1^Working group "Cellular Interactions of Bacterial Pathogens", ZBS 2, Robert Koch Institute, Berlin, Germany

^2^Charité - Universitätsmedizin Berlin, Corporate Member of Freie Universität Berlin and Humboldt-Universität zu Berlin, Department of Infectious Diseases, Respiratory Medicine and Critical Care, Berlin, Germany

^3^Advanced Light and Electron Microscopy, ZBS4, Robert Koch Institute, Berlin, Germany

^4^Bernhard Nocht Institute for Tropical Medicine, Hamburg, Germany

^5^Department for General and Thoracic Surgery, DRK Clinics, Berlin, Germany

† These authors contributed equally to this work and share first authorship

*** Correspondence:**Klaus Heuner
heunerk@rki.de

Stefan Hippenstiel
stefan.hippenstiel@charite.de


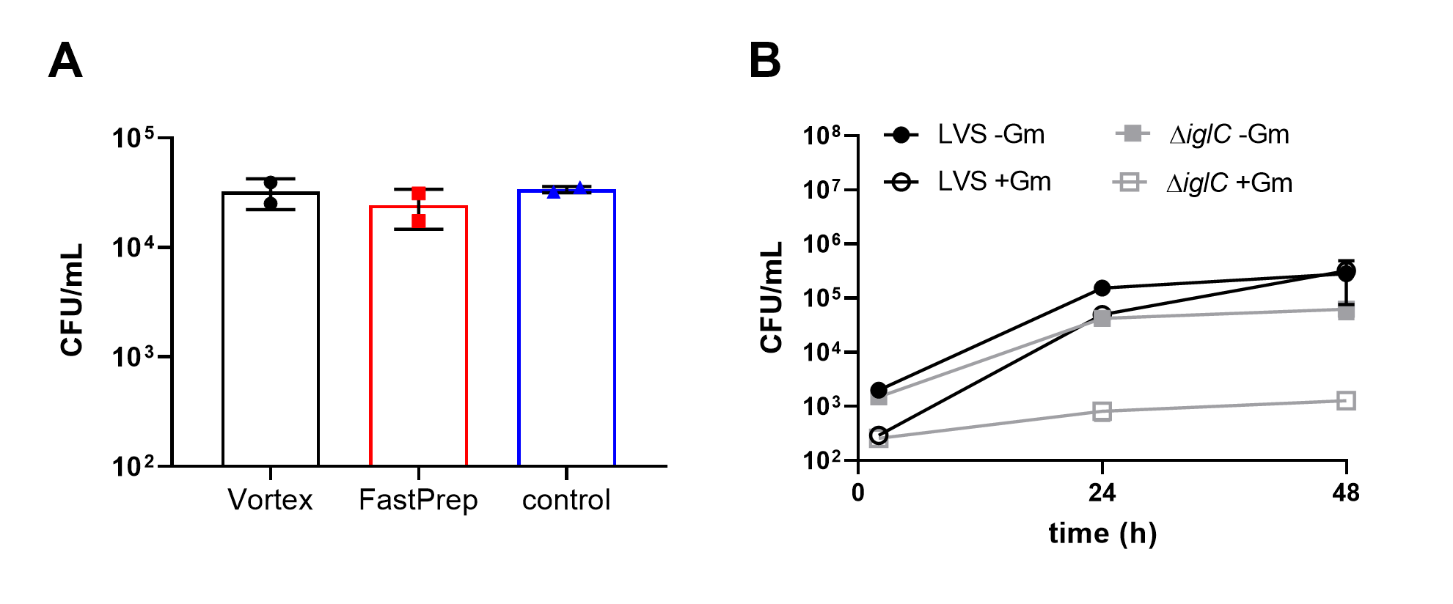


**Supplementary Figure 1.** Pre-tests. **A**: Lysing Matrix D supplemented with saponin were inoculated with *Fth* LVS bacteria and were treated with Vortex shaker for 20 sec or with FastPrep homogenizer with 4 m/s for 20 sec or left untreated (control). Colony forming units per mL (CFU/mL) were determined by plating bacterial suspension onto MTKH agar plates. n = 2. **B**: Infection of human macrophage-like cell line U937. Macrophages were infected with *Fth* LVS and *Fth* LVS ∆*iglC* for 2 h (with a multiplication of infection of 10) in RMPI supplemented with 10% FCS. Bacterial suspension was removed, cells were washed and fresh RMPI + 10% FCS were added. The CFU/mL was determined by lysing the cells with saponin and plating suspension onto agar plates after 24 h and 48 h of incubation; n = 2.


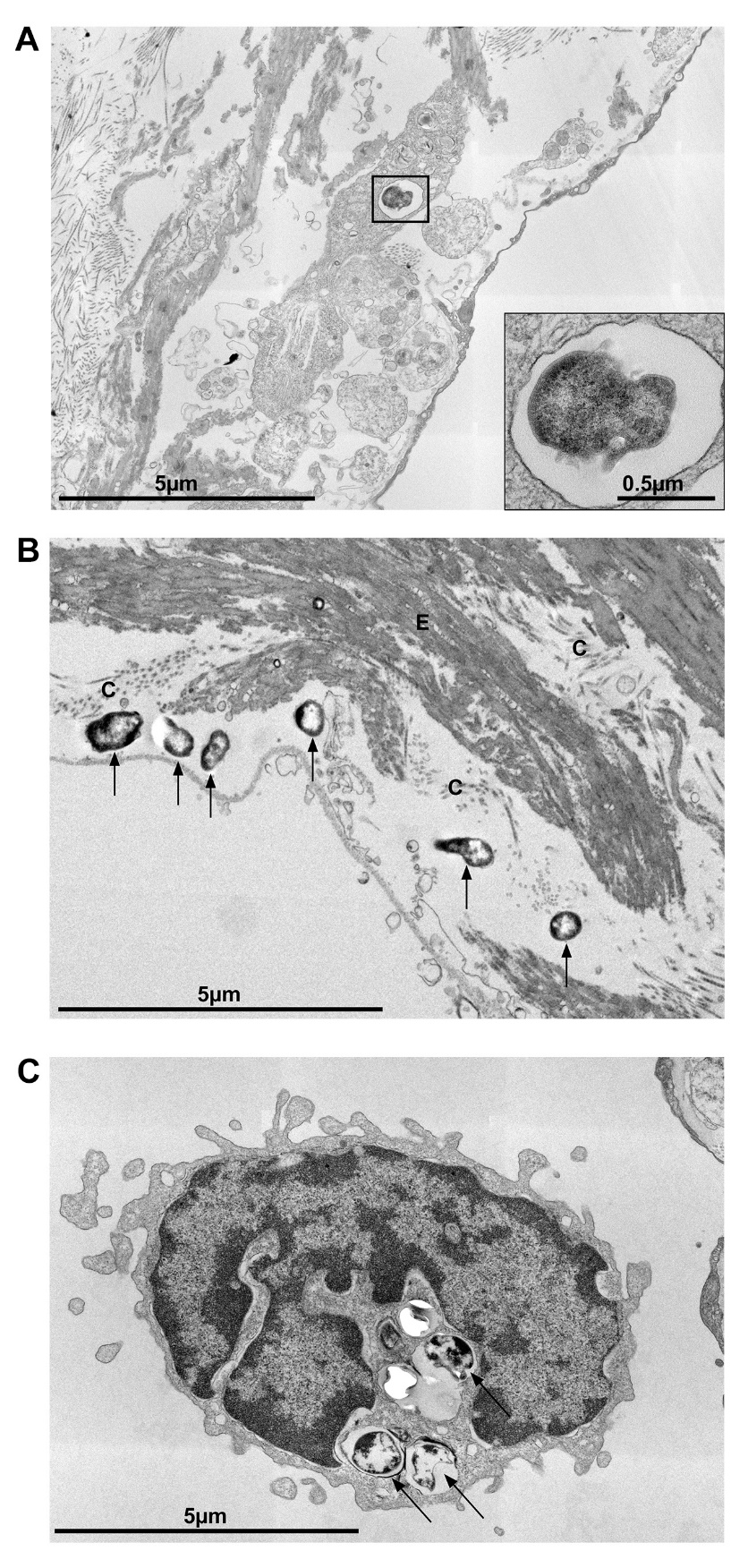


**Supplementary Figure 2:** Electron microscopy of thin sections through human lung tissue explants infected with *Fth* LVS A-660. A: A single profile of a *Fth* A-660 bacterium in a membrane-bound compartment of the cytoplasm of a putative fibrocyte. B: Several extracellular *Fth* A‑660 bacteria in the connective tissue of an alveolar septum (Arrow: Bacteria, C: Collagen, E: elastic fibres). C: A lymphocyte which contains at least three *Fth* A‑660 bacteria in individual membrane-bound compartments.


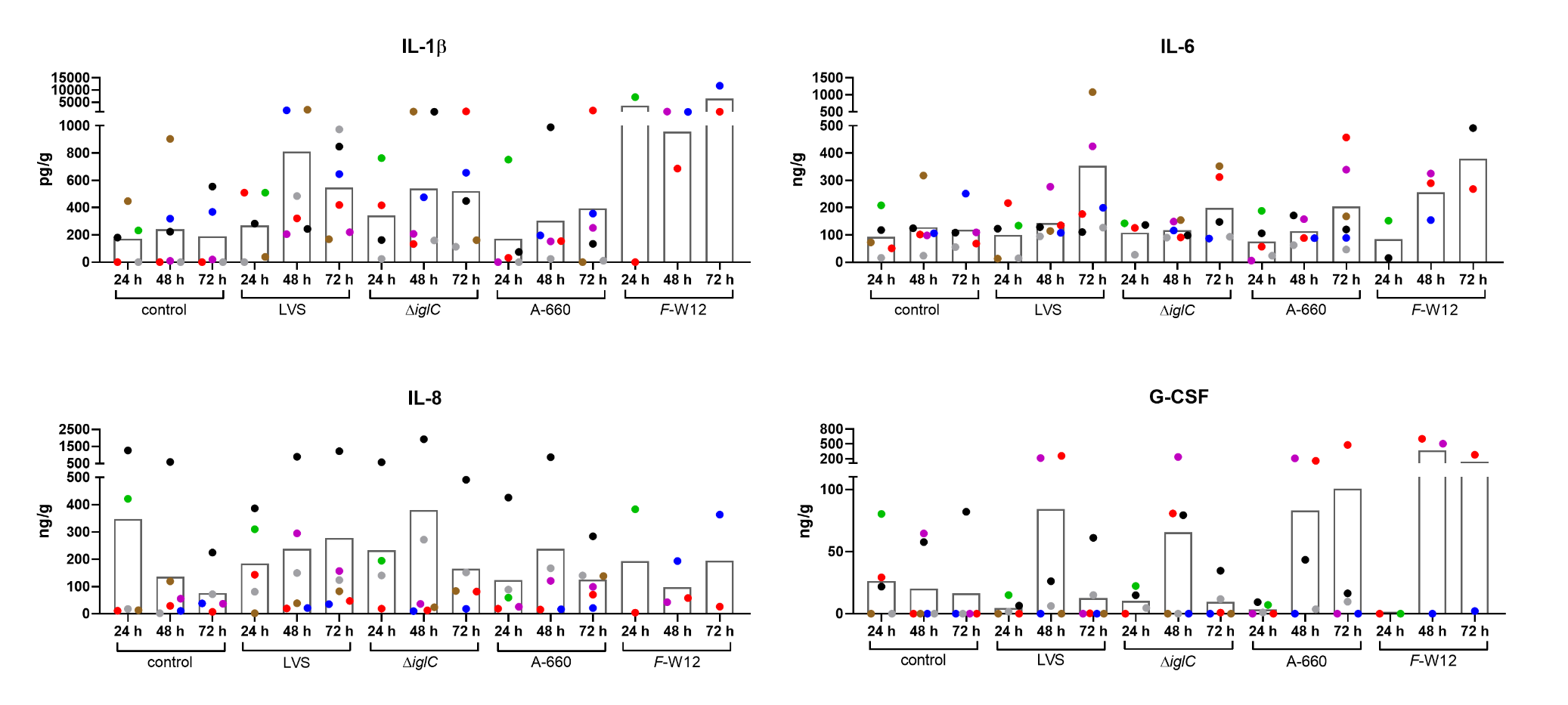


**Supplementary Figure 2_1:** Concentration of several cytokines and chemokines obtained using the *Francisella* human lung *ex vivo* infection model. Lung tissue explants were infected with *Fth* A-660, *Fth* LVS, *Fth* LVS ∆*iglC* and *F*-W12 for 2 h, treated with Gm for 1 h (see material and methods, Fig. 1 and Fig. 2). After 24 h, 48 h and 72 h of infection the supernatants were collected and the concentration of cytokines and chemokines were measured. Each colored dot represents cytokine or chemokine level of an individual donor.


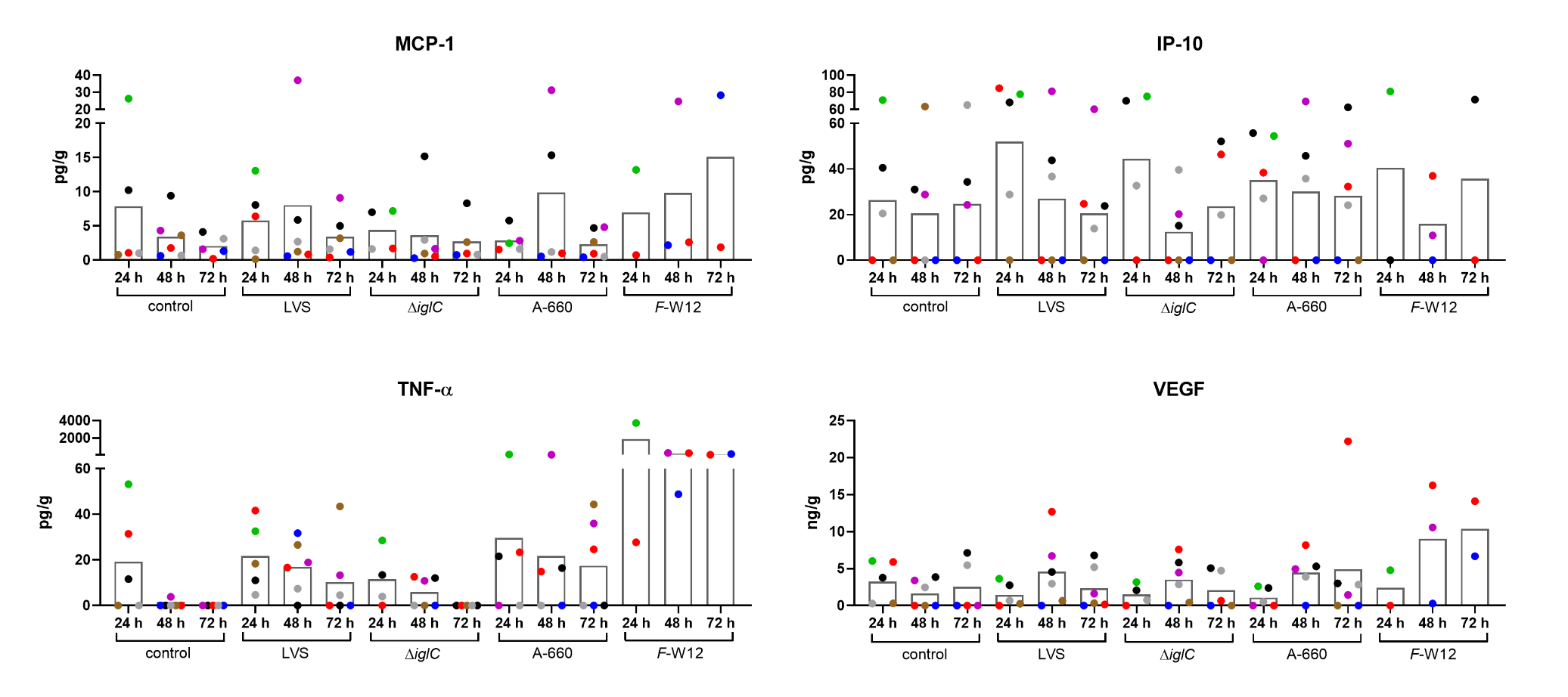


**Supplementary Figure 2_2:** Concentration of several cytokines and chemokines obtained using the *Francisella* human lung *ex vivo* infection model. Lung tissue explants were infected with *Fth* A-660, *Fth* LVS, *Fth* LVS ∆*iglC* and *F*-W12 for 2 h, treated with Gm for 1 h (see material and methods, Fig. 1 and Fig. 2). After 24 h, 48 h and 72 h of infection the supernatants were collected and the concentration of cytokines and chemokines were measured. Each colored dot represents cytokine or chemokine level of an individual donor.
